# Does Vegetation Recovery Limit the Habitat Use of Herbivore? Decadal Evidence of a Potential Ecological Mismatch

**DOI:** 10.3390/biology15060491

**Published:** 2026-03-19

**Authors:** Zhiwei Liu, Zhangfeng Cheng, Rui Guo, Qian Lei, Liulin Guan, Xiao Song, Shanshan Zhao, Aichun Xu

**Affiliations:** 1 College of Life Sciences, China Jiliang University, 258 Xueyuan Rd., Hangzhou 310018, China; 2 Zhejiang Qingliangfeng National Nature Reserve Administration, Hangzhou 311300, China

**Keywords:** deer, ecological restoration, forest ecosystems, decadal camera-trapping

## Abstract

Large-scale forest ecological restoration is commonly expected to improve habitat quality and promote population growth of forest-dependent herbivores. Yet, whether vegetation recovery facilitates or constrains herbivore growth and habitat use at local scales within nature reserves remains unclear, as vegetation recovery and canopy closure might alter forage availability and lead to ecological mismatch between vegetation features and population dynamic. Here, we used the endangered species South China sika deer as the study species, and its dominant distribution region—Qingliangfeng Biosphere Reserve—as the study area. Using decadal camera-trapping data (2015–2024) and extracted vegetation and other environmental variables, we quantified decadal trends in sika deer activity intensity and interannual variation in vegetation (leaf area index, LAI, and normalized difference vegetation index, NDVI). We incorporated topographic and anthropogenic disturbance variables and applied generalized linear mixed models and generalized linear models to analyze its habitat use. We found that: (1) Numbers of independent photographs and the relative abundance index of sika deer increased significantly and consistently from 2015 to 2024. (2) LAI exhibited substantial interannual variability without a stable trend. In contrast, segmented regression identified a clear temporal breakpoint in NDVI, with a significant increasing trend before 2021 followed by a pronounced decline thereafter. (3) In all years, distance to settlement had a significant and negative effect on activity intensity, whereas distance to road, elevation, and year had significant positive effects. LAI and NDVI showed negative and weak effects on sika deer activity intensity. In specific years, LAI had a significantly negative effect in early periods whereas NDVI became significantly negative in mid and late periods. Other environmental variables exhibited interannual heterogeneity. Our findings demonstrate that vegetation recovery within the reserve does not automatically improve habitats for forest-dependent herbivores and could lead to a potential ecological mismatch.

## Simple Summary

In the context of large-scale forest ecological restoration, we examined the relationship between decadal (2015–2024) activity intensity of sika deer and vegetation (leaf area index and normalized difference vegetation index), together with topographic and anthropogenic factors. We found that activity intensity of sika deer had a sustained increase over time. Vegetation indices had weak, periodical effects on sika deer activity, whereas topographic and anthropogenic variables exhibited significant effects. Our findings demonstrate that vegetation recovery within the reserve does not automatically improve habitats for forest-dependent herbivores, and could lead to a potential ecological mismatch.

## 1. Introduction

Large-scale ecological restoration and conservation initiatives have accelerated forest recovery globally over recent decades [1]. In China, the Natural Forest Protection Program (NFPP) and the expansion of the protected-area network have markedly improved forest ecosystem conditions. By 2018, the NFPP had increased national forest cover from 12.7% in 1973 to 22.96%, while total forest growing stock rose from 8.7 billion m^3^ to 17.6 billion m^3^ [2]. As one of the world’s largest restoration programs, the Natural Forest Protection Program has substantially reduced large-scale logging in natural forests [3]. These policy interventions have created favorable conditions for the recovery and expansion of large herbivore populations.

However, vegetation recovery does not necessarily lead to parallel improvements in local habitat quality for large herbivores [4]. Increases in ecological mismatches between vegetation recovery and large herbivores might occur. In the early stage of vegetation recovery, the increase in food resources promotes the growth of herbivore populations and allows them to reach a peak. In the middle and late stages of vegetation recovery, progressive canopy closure reduces understory light availability, thereby suppressing the growth and regeneration of herbaceous and shrub layers [5]. These changes may limit understory foraging and constrain herbivores that depend on understory vegetation [6]. In addition, as vegetation recovery and succession progress, plant communities in some shrub-dominated habitats may become excessively dense, forming closed shrub thickets that restrict the movement space of large herbivores [7]. Therefore, it is vital to identify the mismatch progress and conduct measures to moderate the paradox for regional sustainable development.

Gaining a deeper understanding of herbivore responses to vegetation recovery requires decadal empirical studies and the explicit evaluation of different vegetation metrics [6,8,9]. Previous research assessing vegetation effects on wildlife has largely relied on short-term monitoring data or single vegetation indices, most commonly the normalized difference vegetation index (NDVI) [10,11,12,13]. NDVI often saturates under closed-canopy and high-biomass conditions [14], limiting its ability to characterize forest canopy structure [15]. In contrast, the leaf area index (LAI) more directly characterizes canopy structural attributes and is widely regarded as an important indicator of forest structural succession [16]. Integrating NDVI and LAI with decadal wildlife activity data offers a more comprehensive approach for characterizing large herbivore responses to vegetation structural change.

Sika deer exhibit a fragmented distribution across East Asia [17]. The South China sika deer (*Cervus nippon kopschi*), an endemic and regionally endangered species in China, is listed as a Class I national protected species and is classified as endangered in the China Biodiversity Red List [18]. At present, this endangered species occurs only in fragmented forest habitats within hilly and mountainous areas of the Zhejiang, Jiangxi, and Anhui provinces [19]. Owing to agricultural, pastoral expansion, forest degradation, and historical poaching pressure, its population remained at extremely low levels for decades, with estimates of approximately 300 individuals by the late 20th century [19,20]. In recent years, strengthened conservation measures and reduced anthropogenic disturbance have facilitated a gradual population recovery. As a typical herbivore, the South China sika deer primarily feeds on shrubs or shrub-grass mosaics, and uses the forests for sheltering [21,22]. It is a kind of wildlife that favors landscapes with low canopy density forests and low-density shrubbery, and avoids the dense cover of forest and shrub [21,22]. A stable population is currently found in the Qingliangfeng Biosphere Reserve. Within this typical subtropical montane forest ecosystem, under intensified conservation management and gradually denser forest and shrub, the South China sika deer provides an opportunity to examine activity intensity in relation to vegetation metrics, as well as topographic and anthropogenic disturbance variables under decadal forest recovery. Therefore, it is a favorable case to illustrate the responses of a large herbivore to vegetation across decadal spatiotemporal scales [23].

Based on camera-trapping monitoring data collected over a decade (2015–2024) and extracted vegetation and other environmental variables from the Qingliangfeng Biosphere Reserve, this study systematically examined the spatiotemporal dynamics of sika deer activity intensity in relation to vegetation succession. Specifically, we aimed to: (1) quantify decadal spatiotemporal trends in sika deer activity intensity; (2) characterize interannual spatiotemporal variation in key vegetation indices; and (3) evaluate the combined effects of vegetation indices and other environmental factors, including topography, hydrology, and anthropogenic disturbance on sika deer habitat use. We hypothesized that: (1) sika deer’s activity intensity and vegetation feature would tend to be dynamic; (2) vegetation and other environmental factors contribute to its habitat use, and ecological mismatch between the activity of deer and vegetation recovery would appear. Our study provides new empirical evidence for understanding vegetation–habitat interactions and habitat use mechanisms in sika deer under ongoing forest succession.

## 2. Materials and Methods

### 2.1. Study Area

**Figure 1:**
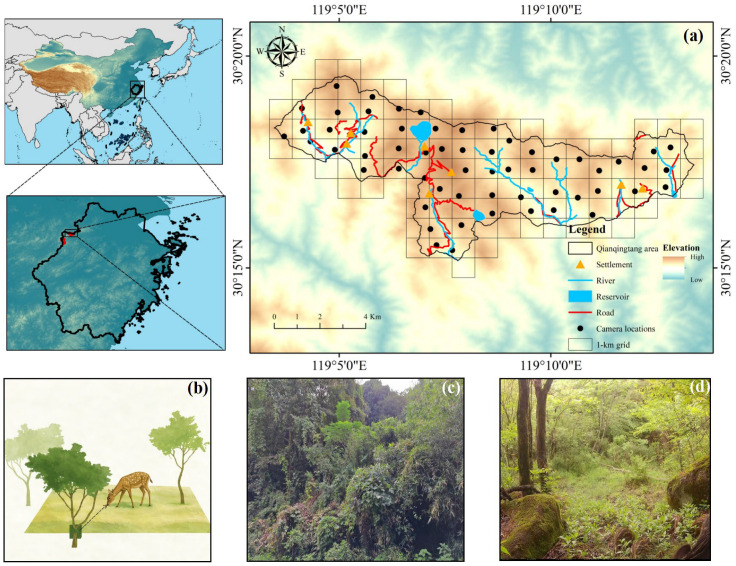
Distribution of the study area, camera-trapping locations, and forest structure in the Qingliangfeng Biosphere Reserve (**a**) Location of the study area and camera-trapping locations (N = 58) in the Qingliangfeng Biosphere Reserve from 2015 to 2024. (**b**) Schematic illustration of camera-trapping-based estimation of sika deer activity intensity. (**c**) Dense forest in the Qingliangfeng Biosphere Reserve. (**d**) Open forest in the Qingliangfeng Biosphere Reserve.

Globally, a total of 785 biosphere reserves have been designated, among which 14 biosphere reserves are distributed in subtropical regions. The Qingliangfeng Biosphere Reserve (30°01′–30°18′ N, 118°50′–119°12′ E) is one of the old reserves in this network and represents a typical subtropical montane forest ecosystem. It is situated in Northwest of Zhejiang, China, and spans an elevation range of 399–1787.4 m [24,25]. The reserve lies at the northern margin of the mid-subtropical zone and has a subtropical monsoon climate [24]. The Qingliangfeng Biosphere Reserve comprises three geographically isolated areas, including Longtangshan, Shunxiwu, and Qianqingtang, among which, the Qianqingtang area constitutes the primary distribution area of the South China sika deer (Figure 1). This area covers approximately 56.9 km^2^ and is characterized by mountainous terrain at elevations of around 1000 m, with relatively gentle slopes and broad, shallow valleys. The mean annual precipitation is approximately 1862 mm, exhibiting strong seasonality, with rainfall concentrated in summer and autumn and comparatively lower precipitation during winter and spring. The mean annual temperature is 11.7 °C, and the accumulated annual temperature ranges from approximately 2200 to 4800 °C, with pronounced variation along elevational gradients and across seasons [26]. Vegetation is dominated by deciduous broad-leaved forests, coniferous forests, and mixed conifer–broad-leaved forests, with localized patches of evergreen broad-leaved forest and meadow habitats, forming a complex mosaic of forest structures and habitat types [27]. The Qingliangfeng Biosphere Reserve supports high biodiversity, with records of 2452 vascular plant species, 355 vertebrate species, and 2567 insect species [25]. This diversity of forest types and topographic conditions provides a highly heterogeneous habitat for forest-dependent herbivores.

### 2.2. Camera-Trapping Survey

Firstly, we divided the study area into 107 square survey grid cells (1 km × 1 km) based on ArcGIS 10.8 (ESRI, Redlands, CA, USA) (Figure 1) [28]. We deployed infrared cameras (Ltl Acorn 6210, Zhuhai Ltl Acorn Electronics Co., Ltd., Zhuhai, China) across the grid system. Cameras were not installed in a small number of grid cells where the protected area was extremely limited. Some camera locations and identification numbers were adjusted during the survey period due to equipment malfunction or inadequate image quality (further details are provided in Table S1). For each grid cell, the geographic center was located in the field using a handheld GPS unit and used as the predefined camera placement point. Cameras were installed within a 50 m × 50 m area centered on this point, preferentially at locations with sparse understory vegetation, along animal trails, or near water sources [29]. Camera placement took local terrain conditions into account, including slope, aspect, and topography. To minimize interference from direct sunlight during sunrise and sunset, cameras were oriented toward the northeast or southwest and mounted on tree trunks at heights of 40–90 cm above ground level. Surrounding environmental conditions at each camera location were also recorded [30]. Prior to deployment, all cameras were configured using standardized settings, including time synchronization, two consecutive photographs per trigger, a minimum trigger interval of 5 s, and medium sensitivity. Cameras were inspected every 4–6 months, during which SD memory cards (32 GB) and batteries were replaced to ensure continuous operation. A total of 58 infrared cameras were deployed in the Qingliangfeng Biosphere Reserve.

We examined retrieved photographs for species identification and recorded associated metadata, including capture time and the number of consecutive images [29,31]. Over the 10-year monitoring period (2015–2024), we accumulated 170,962 camera-trapping days and recorded 15,614 independent photographs. We defined independent photographs as consecutive images of the same species at the same camera location in each 30 min [31]. And pictures of the same species within 30 min were recorded as 1 independent photograph.

### 2.3. Explanatory Variables

Previous studies have demonstrated that topographic features, vegetation indices, and distance to water sources significantly influence habitat use by sika deer [21,22,32]. Accordingly, we considered four categories of explanatory variables, including topography, vegetation, hydrology, and anthropogenic disturbance (Table 1). Elevation and slope were derived from a digital elevation model (GDEMV3, 30 m resolution) using spatial analysis in ArcGIS 10.8. Vector data for the road network, settlements, and hydrological features were initially obtained from the National Geomatics Center of China (www.webmap.cn). To improve spatial accuracy, we manually corrected and supplemented these vector datasets in ArcGIS 10.8 using high-resolution satellite imagery, allowing a more realistic representation of current patterns of human activity and hydrological distribution within the study area. We then used Euclidean distance tools to calculate the distance from each sampling unit to the nearest water body and settlement.

We used the LAI to represent total leaf area per unit ground surface area as an indicator of canopy structural characteristics [33] and the NDVI to characterize vegetation greenness and reflect vegetation growth activity [34]. LAI data were obtained from the MODIS MCD15A3H product (4-day composite, 500 m spatial resolution), and annual mean values were calculated using Google Earth Engine. NDVI data were derived from the MODIS MOD13Q1 product (16-day composite, 250 m spatial resolution) and were similarly processed to obtain annual mean values [35]. We calculated annual averages of both indices for each year from 2015 to 2023.

### 2.4. Statistical Analyses

We analyzed spatiotemporal pattern dynamics of sika deer in the Qingliangfeng Biosphere Reserve. To examine temporal trends in sika deer activity intensity from 2015 to 2024, we fitted generalized linear mixed models (GLMMs) with year as a fixed effect, the number of independent photographs and the relative abundance index separately as response variables [36]. Camera ID was incorporated as a random effect to account for repeated measurements across sampling locations. Prior to model fitting, we evaluated the distributional properties of the response variables by examining the relationship between the mean and variance (Table S1). Because the variance exceeded the mean (Table S1), we used a negative binomial distribution to account for overdispersion. We compared the trend model (including year) with a null model (excluding year) using AICc, and likelihood ratio test (LRT) [36]. Spatially, we applied the kernel density estimation to visualize the spatial distribution of sika deer within the Qingliangfeng Biosphere Reserve for each year.

We analyzed spatiotemporal dynamics of vegetation indices in the Qingliangfeng Biosphere Reserve. Temporally, we used one-way ANOVA to analyze annual grid level data to test for interannual differences in the LAI and the NDVI. In this analysis, LAI and NDVI extractions were based on the 1 km × 1 km grid cells, and we have calculated annual LAI and NDVI for each of the 107 grid cells across the full study period (2015–2024). In addition, to further examine decadal temporal trends in vegetation indices overall in the study area, we applied segmented linear regression to analyze decadal changes in vegetation indices [37,38]. Spatially, we fitted ordinary least squares regressions to grid-level LAI and NDVI time series (2015–2024) and extracted the interannual trend slope for each of the 107 grid cells. We used these slopes to quantify spatial variation in both the direction and magnitude of vegetation change across the study area. We then applied kernel density estimation to visualize the annual spatial patterns of vegetation change within the Qingliangfeng Biosphere Reserve.

To examine the effects of vegetation and other environmental factors on habitat use by the South China sika deer, we employed GLMMs and generalized linear models (GLMs) [39,40]. We conducted the all-year analysis and specific-year analysis. The response variable was the number of independent photographs of sika deer. Because the data were count-based and exhibited overdispersion (Table S1), we fitted all models using a negative binomial distribution implemented via the glm.nb function in the MASS package (version 7.3-65) in R (version 4.5.2) [41]. During all-year analysis, explanatory variables included elevation, slope, NDVI (or LAI), and distances to settlements, water sources, roads, and year. Due to potential collinearity between NDVI and LAI [42,43], two suits of models involving NDVI or LAI were created. Camera ID was served as random effect [44]. During specific-year analysis, we excluded the year and conducted GLMs. We also applied the negative binomial distribution because the overdispersion parameters (ĉ) lied 0.950–1.642 based on the assumed models with negative binomial distribution (Table S2). In addition, we generated candidate models and performed model selection based on Akaike’s information criterion in all-year and specific-year analysis. Models with ΔAICc < 4 were treated as the optimal models, and retained for model averaging because this threshold captured most of the cumulative Akaike weight [45,46]. Parameter estimates were then obtained using model-averaged coefficients across the optimal models [47]. In addition, nested models within optimal models were further compared with the best model using LRT to assess whether inclusion or exclusion environmental variables significantly could improve the model fit.

## 3. Results

### 3.1. Spatiotemporal Patterns of Sika Deer

**Figure 2:**
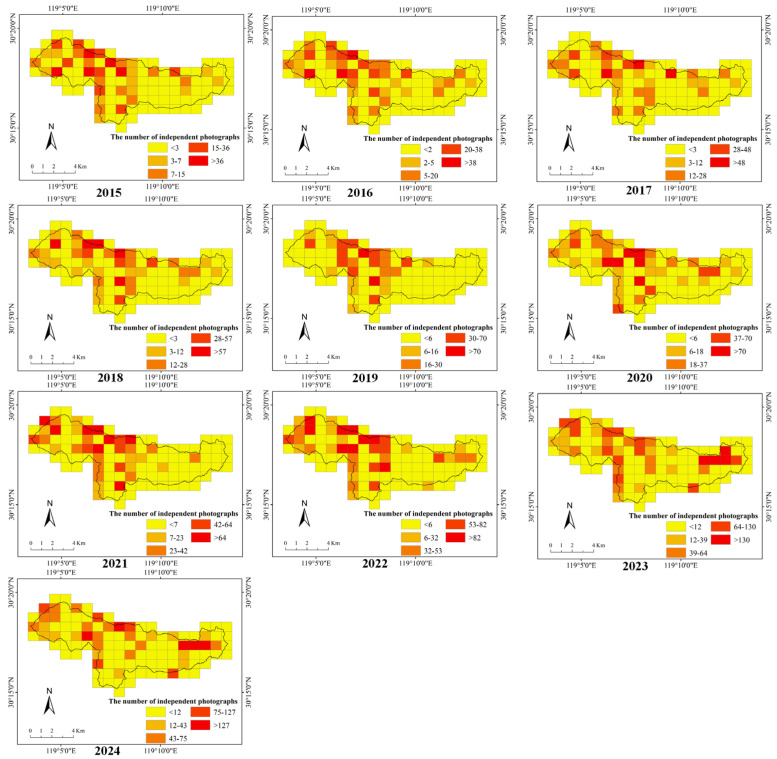
Spatial distribution of the number of independent photographs of sika deer in the Qingliangfeng Biosphere Reserve, 2015–2024.

From 2015 to 2024, we accumulated 170,962 camera-trapping days and recorded 15,614 independent photographs. Time-series analyses based on camera-trapping monitoring data revealed a consistent and statistically significant increase over time in both the number of independent photographs and the relative activity index of sika deer (Figure 2 and Figure S1, Tables S3 and S4). The LRT results showed that the model including the year significantly improved model fit (χ^2^ = 176.28, *p* < 0.001) (Table S3). Year had a significant positive effect (Estimate = 0.18 ± 0.012 SE, z = 14.69, *p* < 0.001) on the number of independent photographs of sika deer (Figure S1, Table S3). A similar increasing trend was detected for the relative abundance index of South China sika deer (Table S4 and Figure S1).

### 3.2. Spatiotemporal Patterns of Vegetation Recovery

**Figure 3:**
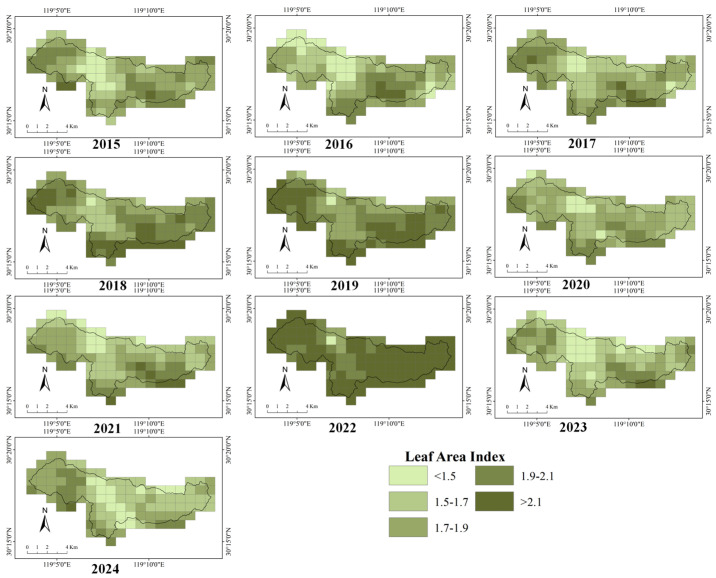
Heatmap of leaf area index in the Qingliangfeng Biosphere Reserve, 2015–2024.

**Figure 4:**
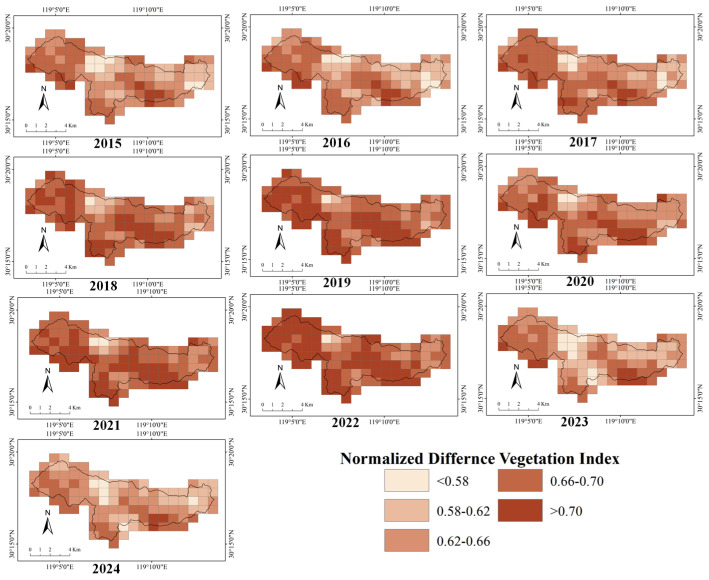
Heatmap of normalized difference vegetation index in the Qingliangfeng Biosphere Reserve, 2015–2024.

The interannual variations in LAI and NDVI at the grid scale within the study area are shown in Figure 3 and Figure 4. One-way ANOVA showed that both LAI and NDVI differed significantly among years within the study area (Tables S5 and S6) (*p* < 0.001), indicating pronounced interannual variation in vegetation indices from 2015 to 2024. Interannual variation in NDVI was relatively small, whereas LAI exhibited greater variability. The temporal changes in the mean values of NDVI and LAI across the study area are shown in Figure S2 and Table S7. Segmented regression analysis indicated that the temporal trend of mean annual NDVI in the study area exhibited a significant breakpoint around 2021.82 (SE = 0.430) (Table S8). Prior to this breakpoint, NDVI increased significantly with year (slope = 0.0067 ± 0.0023, *t* = 2.88, *p* = 0.028). In contrast, NDVI declined significantly after the breakpoint (slope = −0.0301 ± 0.009, *t* = −3.510, *p* < 0.010). The segmented model showed a good fit, explaining a substantial proportion of the interannual variation in NDVI (adjusted R^2^ = 0.679). By contrast, segmented regression analysis did not identify a significant temporal breakpoint in mean annual LAI over the study period (Table S9). Although the estimated breakpoint occurred in 2018.73, its associated standard error was large (SE = 3.710). The slopes before and after the breakpoint were both non-significant (before breakpoint: slope = 0.050 ± 0.097, *t* = 0.52, *p* = 0.624; after breakpoint: slope = −0.030 ± 0.052, *t* = −0.57, *p* = 0.097). Overall, the segmented model exhibited poor explanatory power (adjusted R^2^ = −0.356).

Spatially, the multi-year distributions of LAI and NDVI at the decadal scale across the study area are shown in Figure S3. Both vegetation indices exhibited clear spatial gradients, with higher LAI and NDVI values primarily distributed along the peripheral areas of the reserve. Overall, the spatial pattern was characterized by higher values in the south than in the north, and in the west than in the east, whereas no clear pattern was observed in the central part of the study area. With respect to interannual trends (Figure S3), the directions of change in LAI and NDVI were spatially inconsistent. Some areas exhibited persistent increases (slope > 0), whereas others showed weak changes or a mixture of positive and negative trends, resulting in an overall patchy spatial pattern.

### 3.3. Vegetation and Other Environmental Variables’ Contribution

Model ranking showed that LAI (or NDVI) fell into the candidate optimal models (Tables S10–S12). LRT results showed that models including LAI or NDVI could not significantly improve model fit compared with the best model (LAI: χ^2^ = 0.744, *p* = 0.388; NDVI: χ^2^ = 0.151, *p* = 0.697) (Tables S11–S13). And model-averaged estimates indicated no significant effects of LAI (Estimate = −0.010 ± 0.027 SE, *p* = 0.721) or NDVI (Estimate = −0.004 ± 0.025 SE, *p* = 0.860) (Table 2). Distance to settlement had a significant and negative effect, whereas distance to road, elevation, and year had significant and positive effects (Table 2). Model including or excluding variables could not improve the model fit significantly (Tables S11–S13).

**Figure 5:**
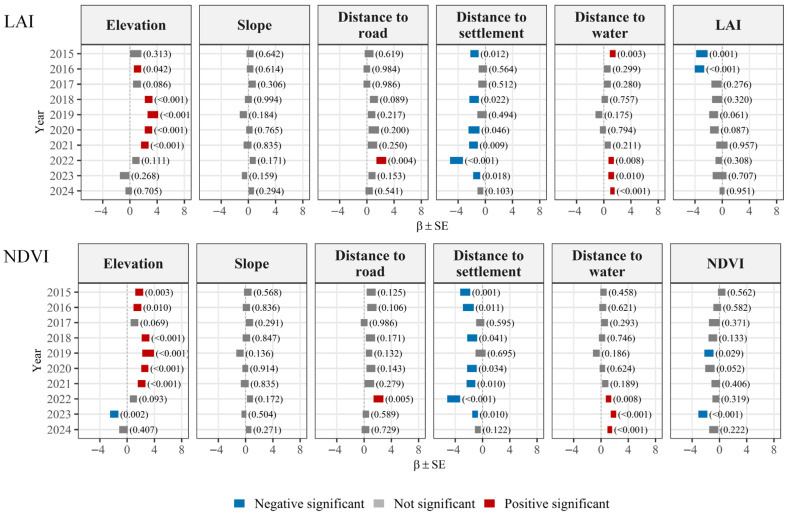
Visualization of generalized linear models regression coefficients describing the relationships between the number of independent photographs of sika deer and explanatory variables in the Qingliangfeng Biosphere Reserve from 2015 to 2024.

LAI (or NDVI) also fell into the candidate optimal models in specific analysis (Tables S14 and S15). Model-averaged results showed that the effects of topographic, hydrological, and anthropogenic variables on sika deer activity intensity exhibited pronounced interannual heterogeneity (Figure 5; Table S16). LAI had a significant and negative effect on deer occurrence in 2015 and 2016 (Figure 5). NDVI showed negative associations with sika deer occurrence in most years except 2015, with a significant negative effect detected in 2019 and 2023.

## 4. Discussion

In the context of vegetation recovery, clarifying the response patterns of herbivores to vegetation characteristics is essential for the conservation of endangered wildlife and for effective habitat management. This study was conducted in the Qingliangfeng Biosphere Reserve and focused on the sika deer as the focal species. By examining the effects of vegetation change on sika deer, this study provides a case example of herbivore responses to vegetation recovery. Vegetation recovery is a key process for alleviating habitat degradation and restoring ecosystem functions. For instance, natural succession and rewilding following farmland abandonment in Europe have facilitated the return and expansion of deer and other large herbivores [48]. In tropical secondary forests, landscapes with higher forest cover have been shown to support greater abundances of ungulates, such as white-tailed deer and collared peccaries [49]. In temperate secondary forests, structural restoration of forest stands can substantially increase deer foraging opportunities and habitat use [50]. These studies indicate that improved habitat conditions constitute a critical ecological basis for the persistence and potential growth of large herbivore populations [48,49,50].

Sika deer activity intensity and vegetation features exhibited interannual heterogeneity, which supports our first hypothesis (both the vegetation and deer’s activity intensity tend to be dynamic over time). At the grid level, vegetation trends within the reserve produced a highly heterogeneous, patchy spatial pattern. Meanwhile, the number of independent photographs of sika deer increased markedly from 2015 to 2024 in the reserve. These patterns contribute to decadal conservation management and reduced anthropogenic disturbance of the reserve. Under the broader framework of the Natural Forest Protection Program, the forests were restricted on logging, and conserved [3]. However, vegetation within the reserve showed pronounced interannual heterogeneity, with no clear overall temporal trend and spatially heterogeneous rates of change (Figure S3). This pattern might be due to the relatively short study period in relation to forest recovery processes, which tends to be insufficient for capturing long-term vegetation trends. Since the establishment of reserve in 1985 [20], the accessibility of residents was restricted, and farming and livestock grazing have decreased [51]. These rules reduced anthropogenic disturbance and contributed to the recovery of vegetation and sika deer’s population.

Vegetation and other environmental factors contributed to deer habitat use in both the all-year and specific-year analyses, partly supporting our second hypothesis (that deer’s habitat use was influenced by environmental factors and a potential ecological mismatch with vegetation recovery emerged). Neither LAI nor NDVI was significantly associated with sika deer activity intensity in all-year analysis. But in specific-year analysis, LAI had a significantly negative effect in early periods whereas NDVI became significantly negative in mid and late periods. The combined results showed an unclear pattern, which suggested obscure deer–forest relations. But negative effects of vegetation indices (NDVI/LAI), although not significant, appeared in most models, which might be the potential ecological mismatch and lie in the primary stage. In addition, due to the small population of sika deer, the abundance of this species is in a rapid growth stage in this region without large carnivorous wildlife. The limitation effects of vegetation have not yet appeared and may exhibit a lag effect [52]. A similar pattern has been reported in other deer studies [53], which proves that density dependence can obscure the relationships between remotely sensed environmental variables and demographic rates. Future research should incorporate the field microhabitat data and seasonal variation associated with vegetation structure to clarify its relationship. And it is necessary to conduct long-term monitoring, identifying the mismatch, and anticipating timely adaptive management.

Elevation, distance to settlement and distance to roads were the key factors influencing the habitat use of sika deer in our study. In all-year analysis and most specific-year analyses, deer tended to inhabit higher elevations in the reserve, and selected the lower elevation habitat in the recent year from 2023 and 2024. This pattern might be due to the density-dependent habitat expansion. In former years, the abundance of deer was small, and the deer prioritized the optimal habitat located in the higher elevation [32]. As the population grew, sika deer shifted to inhabit in the low elevation region [20,22]. Sika deer selected the near settlement regions, which could be explained by the special spatial distribution of settlements. The settlements in the reserve are protection stations [20,54], which could not produce threats for deer. Sika deer avoided roads, consistent with widespread evidence that roads function as high-risk landscape features that constrain ungulate movement and habitat use through persistent anthropogenic disturbance [55,56,57]. Overall, it is necessary to partition the conservation zone in relation to elevation, settlements and roads, and conduct specific measures for sustainable development.

## 5. Limitations

Some limitations occurred in current study. We focused on the independent photographs of sika deer and explored its relationships with the vegetation. However, other aspects, such as group size, the use and dynamics of trails, interspecific relationships among herbivores, and browsing impacts of herbivores on the forest, were not included, which were directly related to vegetation structure and should be investigated in the future.

## 6. Conclusions

Using camera-trapping data collected over a decade (2015–2024) and associated with vegetation and environmental factors extracted for the Qingliangfeng Biosphere Reserve, this study systematically examined the spatiotemporal dynamics of sika deer activity intensity in relation to vegetation succession. We found that both the number of independent photographs and the relative abundance index of sika deer show a sustained increase over time. Vegetation indices showed weak, period-dependent effects on sika deer activity, whereas anthropogenic variables exhibited clearer effects. We recommend that long-term monitoring of sika deer and investigation of its habitat and microhabitat should be enhanced. Our findings provide new empirical evidence for understanding the habitat use of large herbivores under forest recovery and offer a scientific basis for forest structural regulation, habitat management, and adaptive management of population dispersal.

## Figures and Tables

**Table 1:** Description of explanatory variables used in the analysis.

Variable	Data Type	Description and Source Source
Hydrological		
Distance to water	Continuous	Euclidean distance to the nearest water, derived from NGCC vector data in ArcGIS 10.8.
Topography		
Elevation	Continuous	Extracted from the 30 m digital elevation model (GDEMV3) in ArcGIS 10.8.
Slope	Continuous	Calculated from the 30 m digital elevation model (GDEMV3) in ArcGIS 10.8.
Vegetation		
Leaf Area Index	Continuous	Annual mean LAI from MODIS MCD15A3H, processed in Google Earth Engine.
Normalized difference vegetation index	Continuous	Annual mean NDVI from MODIS MOD13Q1, processed in Google Earth Engine.
Anthropogenic disturbance		
Distance to road	Continuous	Euclidean distance to the nearest road, derived from NGCC vector data in ArcGIS 10.8.
Distance to settlement	Continuous	Euclidean distance to the nearest settlement, derived from NGCC vector data in ArcGIS 10.8

NGCC, National Geomatics Center of China; GDEMV3, Global Digital Elevation Model version 3 (30 m spatial resolution); MODIS MCD15A3H, MODIS Leaf Area Index product (4-day composite, 500 m spatial resolution); MODIS MOD13Q1, MODIS vegetation indices product providing NDVI (16-day composite, 250 m spatial resolution).

**Table 2:** Model-averaged results in examining the relationships between the number of independent photographs of sika deer and explanatory variables from 2015 to 2024. Separate models were constructed for leaf area index and normalized difference vegetation index. Explanatory variables included leaf area index, normalized difference vegetation index, distance to road, distance to settlement, distance to water, elevation, slope, and year.

Models	Variable	Estimate	SE	z Value	*p*
NDVI	Intercept	2.184	0.104	21.068	<0.001
	Distance to road	0.242	0.120	2.017	0.044
	Distance to settlement	−0.413	0.098	4.220	<0.001
	Distance to water	0.192	0.116	1.657	0.097
	Elevation	0.233	0.114	2.039	0.041
	Year	0.177	0.012	14.337	<0.001
	Slope	0.007	0.036	0.202	0.840
	NDVI	−0.004	0.025	0.176	0.860
LAI	Intercept	2.184	0.103	21.110	<0.001
	Distance to road	0.241	0.119	2.030	0.042
	Distance to settlement	−0.413	0.097	4.239	<0.001
	Distance to water	0.194	0.115	1.690	0.091
	Elevation	0.232	0.113	2.044	0.041
	Year	0.177	0.012	14.382	<0.001
	LAI	−0.010	0.027	0.357	0.721
	Slope	0.007	0.036	0.202	0.840

## Data Availability

The original contributions presented in this study are included in the article/Supplementary Materials. Further inquiries can be directed to the corresponding authors.

## Supplementary Materials

The following supporting information can be downloaded at: https://www.mdpi.com/article/10.3390/biology15060491/s1, Figure S1: Predicted temporal trends in the number of independent photographs and relative abundance index of South China sika deer from 2015 to 2024. Solid lines represent model predictions and shaded areas indicate 95% confidence intervals; Table S1: The number of independent photographs and the relative abundance index of sika deer across camera-trappings in the Qingliangfeng Biosphere Reserve from 2015 to 2024; Table S2: Overdispersion parameter (ĉ) for year-specific generalized linear models with a negative binomial distribution examining relationships between the number of independent photograph of sika deer and explanatory variables (distance to road, distance to settlement, distance to water, elevation, slope, and Leaf Area Index (Normalized Difference Vegetation Index) from 2015 to 2024; Table S3: Results of the Generalized Linear Mixed Model testing temporal trends in number of independent photographs of sika deer from 2015 to 2024; Table S4: Results of the Generalized Linear Mixed Model testing temporal trends in relative abundance index of South China sika deer from 2015 to 2024; Table S5: Results of one-way ANOVA testing interannual variation in Leaf Area Index in relation to South China sika deer in the Qingliangfeng Biosphere Reserve from 2015 to 2024; Table S6: Results of one-way ANOVA testing interannual variation in Normalized Difference Vegetation Index in relation to South China sika deer in Qingliangfeng Biosphere Reserve from 2015 to 2024; Figure S2: Interannual variation in annual mean Leaf Area Index and Normalized Difference Vegetation Index in the Qingliangfeng Biosphere Reserve from 2015 to 2024; Table S7: Annual mean Normalized Difference Vegetation Index and Leaf Area Index in the Qingliangfeng Biosphere Reserve from 2015 to 2024; Table S8: Result of segmented regression analysis of temporal changes in annual mean Normalized Difference Vegetation Index in the Qingliangfeng Biosphere Reserve from 2015 to 2024; Table S9: Result of segmented regression analysis of temporal changes in annual mean Leaf Area Index in the Qingliangfeng Biosphere Reserve from 2015 to 2024; Figure S3: Spatial patterns of multi-year mean Leaf Area Index and Normalized Difference Vegetation Index, and spatial distribution of their interannual trends in the Qingliangfeng Biosphere Reserve. (a) Spatial distribution of multi-year Leaf Area Index; (b) Spatial distribution of multi-year Normalized Difference Vegetation Index; (c) Spatial distribution of interannual trends in Leaf Area Index, represented by linear regression slopes; (d) Spatial distribution of interannual trends in Normalized Difference Vegetation Index, represented by linear regression slopes; Table S10: AICc-based model ranking results in examining the relationships between the number of independent photographs of sika deer and explanatory variables from 2015 to 2024. Candidate models were derived from the global model including distance to road, distance to settlement, distance to water, elevation, slope, year, and Normalized Difference Vegetation Index; Table S11: Likelihood ratio tests comparing nested candidate models with the best model (Independent Photographs ~ Distance to road + Distance to settlement + Distance to water + Elevation + Year); Table S12: AICc-based model ranking results in examining the relationships between the number of independent photographs of sika deer and explanatory variables from 2015 to 2024. Candidate models were derived from the global model including distance to road, distance to settlement, distance to water, elevation, slope, year, and Leaf Area Index; Table S13: Likelihood ratio tests for comparing nested candidate models with the best model (Independent Photographs ~ Distance to road + Distance to settlement + Distance to water + Elevation + Year); Table S14: Results of year-specific candidate models with ΔAICc < 4 assessing the relationships between independent photograph counts of sika deer and explanatory variables (distance to road, distance to settlement, distance to water, elevation, slope, and Leaf Area Index) from 2015 to 2024; Table S15: Results of year-specific candidate models with ΔAICc < 4 assessing the relationships between independent photograph counts of sika deer and explanatory variables (distance to road, distance to settlement, distance to water, elevation, slope, and Normalized Difference Vegetation Index) from 2015 to 2024; Table S16: Model-averaged results for exploring the relationships between numbers of independent photographs of sika deer and explanatory variables (distance to road, distance to settlement, distance to water, elevation, slope, year, Normalized Difference Vegetation Index, and Leaf Area Index) from 2015 to 2024. Leaf Area Index and Normalized Difference Vegetation Index were modeled separately in optimal models. Models with ΔAICc < 4 were selected and averaged.
