# Supplementary material for: Does Vegetation Recovery Limit the Habitat Use of Herbivore? Decadal Evidence of a Potential Ecological Mismatch
Source: Biology (Basel). 2026 Mar 19;15(6):491. doi: 10.3390/biology15060491 (PMC13023506; doi:10.3390/biology15060491)
Supplement: Supplementary file 1 [file biology-15-00491-s001.zip › biology-4160522-supplementary.pdf]

Supplementary Materials

Figure S1. Predicted temporal trends in the number of independent photographs and relative abundance index of South China sika deer from 2015 to 2024. Solid lines represent model predictions and shaded areas indicate 95% confidence intervals.

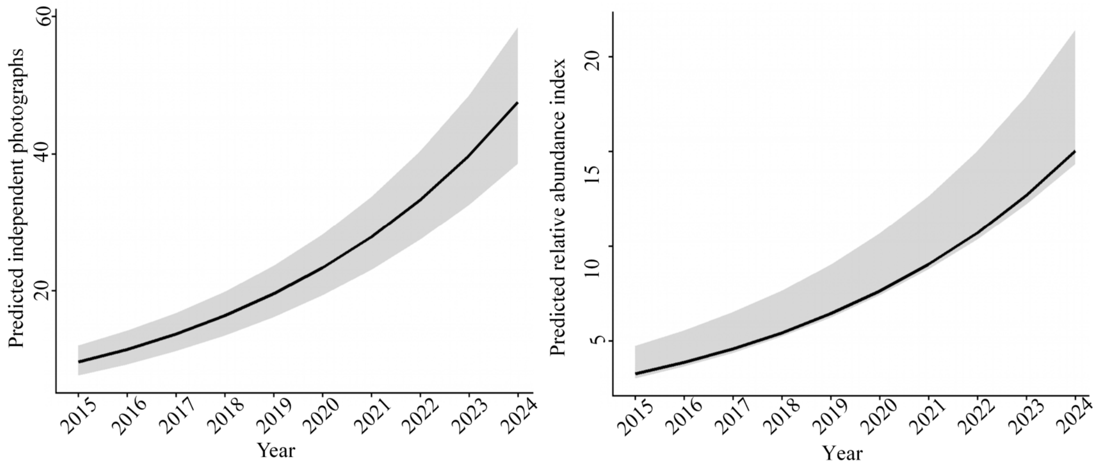

Table S1. The number of independent photographs and the relative abundance index of sika deer across camera-trappings in the Qingliangfeng Biosphere Reserve from 2015 to 2024.

Year	Camera ID	IP	RAI
2024	1	69	18.90
2024	2	51	13.97
2024	3	36	9.86
2024	4	38	10.41
2024	5	33	9.04
2024	7	49	13.42
2024	8	47	12.88
2024	9	46	12.60
2024	10	12	3.29
2024	12	41	11.23
2024	15	180	49.32
2024	16	45	12.33
2024	18	83	22.74

2024	19	45	12.33
2024	20	48	13.15
2024	22	27	7.40
2024	23	30	8.22
2024	25	12	3.29
2024	27	20	5.48
2024	28	32	8.77
2024	29	12	3.29
2024	30	12	3.29
2024	32	195	53.42
2024	33	103	28.22
2024	34	69	18.90
2024	35	12	3.29
2024	36	75	20.55
2024	37	12	3.29
2024	40	72	19.73
2024	41	28	7.67
2024	43	12	3.29
2024	44	12	3.29
2024	46	21	5.75
2024	47	21	5.75
2024	48	170	46.58
2024	49	43	11.78
2024	50	192	52.60
2024	51	12	3.29
2024	57	86	23.56
2024	58	58	15.89
2024	A1	128	35.07
2024	A2	44	12.05
2024	A3	31	8.49
2024	C1	62	16.99
2024	D1	156	42.74
2024	D2	30	8.22
2024	D3	99	27.12
2024	D4	117	32.05
2024	Z4	47	12.88
2024	Z5	68	18.63
2023	1	34	9.32
2023	2	29	7.95
2023	3	33	9.04
2023	4	26	7.12

2023	5	53	14.52
2023	7	88	24.11
2023	8	79	21.64
2023	9	36	9.86
2023	10	18	4.93
2023	12	24	6.58
2023	15	110	30.14
2023	16	55	15.07
2023	17	131	35.89
2023	18	99	27.12
2023	19	65	17.81
2023	20	22	6.03
2023	21	87	23.84
2023	22	48	13.15
2023	23	28	7.67
2023	24	46	12.60
2023	25	18	4.93
2023	26	6	1.64
2023	27	39	10.68
2023	28	23	6.30
2023	29	6	1.64
2023	30	101	27.67
2023	32	98	26.85
2023	33	48	13.15
2023	34	36	9.86
2023	35	24	6.58
2023	36	60	16.44
2023	37	6	1.64
2023	39	24	6.58
2023	40	12	3.29
2023	41	24	6.58
2023	42	16	4.38
2023	50	175	47.95
2023	51	60	16.44
2023	52	12	3.29
2023	55	156	42.74
2023	57	224	61.37
2023	58	99	27.12
2023	A1	81	22.19
2023	A2	30	8.22
2023	A3	27	7.40

2023	C1	30	8.22
2023	D1	207	56.71
2023	D2	43	11.78
2023	D3	84	23.01
2023	D4	99	27.12
2023	Z1	6	1.64
2023	Z2	50	13.70
2023	Z4	33	9.04
2023	Z5	35	9.59
2023	Z6	26	7.12
2022	1	76	20.82
2022	2	36	9.86
2022	3	19	5.21
2022	4	14	3.84
2022	5	53	14.52
2022	7	93	25.48
2022	8	105	28.77
2022	9	20	5.48
2022	10	19	5.21
2022	12	27	7.40
2022	15	86	23.56
2022	16	26	7.12
2022	17	96	26.30
2022	18	110	30.14
2022	19	116	31.78
2022	20	105	28.77
2022	21	34	9.32
2022	22	43	11.78
2022	23	23	6.30
2022	24	37	10.14
2022	25	32	8.77
2022	26	2	0.55
2022	27	17	4.66
2022	28	67	18.36
2022	29	73	20.00
2022	30	63	17.26
2022	32	87	23.84
2022	33	16	4.38
2022	34	82	22.47
2022	35	36	9.86
2022	36	100	27.40

2022	37	5	1.37
2022	41	14	3.84
2022	42	15	4.11
2022	43	4	1.10
2022	50	27	7.40
2022	51	4	1.10
2022	54	2	0.55
2022	55	20	5.48
2022	57	64	17.53
2022	58	39	10.68
2022	A1	28	7.67
2022	A2	51	13.97
2022	A3	50	13.70
2022	C1	67	18.36
2022	D1	37	10.14
2022	D2	22	6.03
2022	D3	19	5.21
2022	D4	21	5.75
2022	Z2	44	12.05
2022	Z4	49	13.42
2022	Z5	16	4.38
2022	Z6	15	4.11
2021	1	49	13.42
2021	2	17	4.66
2021	3	9	2.47
2021	4	7	1.92
2021	5	20	5.48
2021	7	72	19.73
2021	8	28	7.67
2021	9	20	5.48
2021	10	11	3.01
2021	11	1	0.27
2021	12	38	10.41
2021	13	3	0.82
2021	15	46	12.60
2021	16	39	10.68
2021	17	70	19.18
2021	18	74	20.27
2021	19	97	26.58
2021	20	75	20.55
2021	21	34	9.32

2021	22	27	7.40
2021	23	28	7.67
2021	24	21	5.75
2021	25	20	5.48
2021	26	2	0.55
2021	27	23	6.30
2021	28	88	24.11
2021	29	64	17.53
2021	30	55	15.07
2021	32	52	14.25
2021	33	27	7.40
2021	34	25	6.85
2021	35	12	3.29
2021	36	23	6.30
2021	37	30	8.22
2021	39	7	1.92
2021	40	12	3.29
2021	41	42	11.51
2021	42	8	2.19
2021	43	3	0.82
2021	44	10	2.74
2021	45	3	0.82
2021	50	39	10.68
2021	51	2	0.55
2021	A1	69	18.90
2021	A2	23	6.30
2021	A3	29	7.95
2021	C1	86	23.56
2021	Z2	3	0.82
2021	Z4	22	6.03
2021	Z5	5	1.37
2021	Z6	7	1.92
2020	1	29	7.95
2020	2	18	4.93
2020	3	9	2.47
2020	4	4	1.10
2020	5	8	2.19
2020	7	47	12.88
2020	8	24	6.58
2020	9	27	7.40
2020	10	24	6.58

2020	12	9	2.47
2020	13	6	1.64
2020	15	74	20.27
2020	16	32	8.77
2020	17	36	9.86
2020	18	8	2.19
2020	19	129	35.34
2020	20	79	21.64
2020	21	11	3.01
2020	22	8	2.19
2020	23	15	4.11
2020	24	17	4.66
2020	25	52	14.25
2020	26	2	0.55
2020	27	27	7.40
2020	28	10	2.74
2020	29	80	21.92
2020	30	79	21.64
2020	32	94	25.75
2020	33	22	6.03
2020	34	15	4.11
2020	35	14	3.84
2020	37	45	12.33
2020	39	1	0.27
2020	40	8	2.19
2020	41	33	9.04
2020	42	28	7.67
2020	43	2	0.55
2020	44	14	3.84
2020	47	12	3.29
2020	48	70	19.18
2020	49	38	10.41
2020	50	60	16.44
2020	54	3	0.82
2020	55	18	4.93
2020	57	33	9.04
2020	58	10	2.74
2019	5	2	0.55
2019	7	71	19.45
2019	8	12	3.29
2019	9	14	3.84

2019	10	12	3.29
2019	12	1	0.27
2019	13	8	2.19
2019	15	50	13.70
2019	16	49	13.42
2019	17	49	13.42
2019	18	55	15.07
2019	19	119	32.60
2019	20	31	8.49
2019	21	48	13.15
2019	22	8	2.19
2019	23	16	4.38
2019	24	6	1.64
2019	25	16	4.38
2019	26	4	1.10
2019	27	2	0.55
2019	28	55	15.07
2019	29	114	31.23
2019	30	50	13.70
2019	31	36	9.86
2019	32	53	14.52
2019	33	20	5.48
2019	34	27	7.40
2019	35	23	6.30
2019	36	16	4.38
2019	37	12	3.29
2019	39	1	0.27
2019	40	21	5.75
2019	41	71	19.45
2019	42	50	13.70
2019	49	12	3.29
2019	51	6	1.64
2018	1	13	3.56
2018	2	2	0.55
2018	3	10	2.74
2018	4	5	1.37
2018	5	12	3.29
2018	7	70	19.18
2018	8	10	2.74
2018	9	15	4.11
2018	10	5	1.37

2018	11	16	4.38
2018	13	12	3.29
2018	15	6	1.64
2018	16	48	13.15
2018	17	90	24.66
2018	18	83	22.74
2018	19	18	4.93
2018	21	5	1.37
2018	22	16	4.38
2018	23	4	1.10
2018	24	3	0.82
2018	25	2	0.55
2018	26	2	0.55
2018	27	10	2.74
2018	28	37	10.14
2018	29	103	28.22
2018	31	58	15.89
2018	32	74	20.27
2018	33	17	4.66
2018	34	14	3.84
2018	35	4	1.10
2018	36	24	6.58
2018	37	18	4.93
2018	39	1	0.27
2018	40	5	1.37
2018	41	40	10.96
2018	42	10	2.74
2018	47	5	1.37
2018	48	6	1.64
2018	49	29	7.95
2018	50	24	6.58
2018	51	3	0.82
2018	55	8	2.19
2018	57	20	5.48
2018	58	4	1.10
2017	1	15	4.11
2017	2	9	2.47
2017	3	15	4.11
2017	4	8	2.19
2017	5	48	13.15
2017	7	48	13.15

2017	9	15	4.11
2017	10	12	3.29
2017	13	2	0.55
2017	15	62	16.99
2017	16	17	4.66
2017	17	46	12.60
2017	18	27	7.40
2017	19	38	10.41
2017	20	26	7.12
2017	21	8	2.19
2017	22	11	3.01
2017	24	15	4.11
2017	25	1	0.27
2017	28	29	7.95
2017	29	21	5.75
2017	31	12	3.29
2017	32	84	23.01
2017	33	5	1.37
2017	34	2	0.55
2017	35	7	1.92
2017	36	21	5.75
2017	37	25	6.85
2017	41	77	21.10
2017	42	24	6.58
2017	43	4	1.10
2017	47	21	5.75
2017	49	14	3.84
2017	54	6	1.64
2017	55	18	4.93
2017	57	6	1.64
2016	1	14	3.84
2016	2	3	0.82
2016	3	3	0.82
2016	4	5	1.37
2016	5	54	14.79
2016	7	35	9.59
2016	8	9	2.47
2016	9	26	7.12
2016	10	9	2.47
2016	11	3	0.82
2016	13	1	0.27

2016	15	73	20.00
2016	16	12	3.29
2016	17	33	9.04
2016	18	72	19.73
2016	19	38	10.41
2016	20	24	6.58
2016	21	14	3.84
2016	22	5	1.37
2016	24	12	3.29
2016	25	4	1.10
2016	27	1	0.27
2016	28	20	5.48
2016	29	33	9.04
2016	31	27	7.40
2016	32	19	5.21
2016	33	12	3.29
2016	34	9	2.47
2016	35	2	0.55
2016	36	5	1.37
2016	37	12	3.29
2016	39	2	0.55
2016	40	4	1.10
2016	41	23	6.30
2016	42	7	1.92
2016	47	12	3.29
2016	48	2	0.55
2016	50	14	3.84
2016	51	2	0.55
2016	54	2	0.55
2016	55	10	2.74
2015	1	10	2.74
2015	2	3	0.82
2015	4	4	1.10
2015	5	44	12.05
2015	6	22	6.03
2015	7	5	1.37
2015	8	18	4.93
2015	9	20	5.48
2015	10	9	2.47
2015	11	2	0.55
2015	13	1	0.27

2015	15	86	23.56
2015	16	12	3.29
2015	17	55	15.07
2015	18	36	9.86
2015	19	52	14.25
2015	20	19	5.21
2015	21	9	2.47
2015	22	19	5.21
2015	23	7	1.92
2015	24	13	3.56
2015	25	6	1.64
2015	26	2	0.55
2015	27	1	0.27
2015	28	20	5.48
2015	29	13	3.56
2015	31	78	21.37
2015	32	11	3.01
2015	33	5	1.37
2015	34	3	0.82
2015	35	7	1.92
2015	36	7	1.92
2015	37	9	2.47
2015	40	2	0.55
2015	41	15	4.11
2015	42	8	2.19
2015	43	1	0.27
2015	44	2	0.55
2015	47	7	1.92
2015	48	14	3.84
2015	49	17	4.66
2015	50	2	0.55
2015	54	5	1.37
2015	55	5	1.37
2015	58	2	0.55

The mean number of independent photographs per camera-year was 34.17 (variance = 1282.57; dispersion index = 37.54), and the mean relative abundance index was 9.36 (variance = 96.27; dispersion index = 10.28), indicating overdispersion in both response variables.

Table S2. Overdispersion parameter (ĉ) for year-specific generalized linear models with a negative binomial distribution examining relationships between the number of independent photograph of sika deer and explanatory variables (distance to road, distance to settlement, distance to water, elevation, slope, and Leaf Area Index (Normalized Difference Vegetation Index) from 2015 to 2024.

Year	NDVI ĉ	LAI ĉ
2015	1.418	1.377
2016	1.512	1.642
2017	1.278	1.224
2018	1.098	1.044
2019	1.150	1.171
2020	1.025	1.085
2021	0.992	0.950
2022	1.187	1.143
2023	1.234	1.306
2024	1.218	1.165

Table S3. Results of the Generalized Linear Mixed Model testing temporal trends in number of independent photographs of sika deer from 2015 to 2024.

Category	Parameter	Estimate	SE	z	χ^2	df	AICc	ΔAICc	*P*
Fixed effects	Intercept	2.08	0.123	16.88					<0.001
	Year	0.18	0.012	14.69					<0.001

Category	Parameter	Estimate	SE	z	χ^2	df	AICc	ΔAICc	*P*
Random effect		Variance: 0.53; Standard deviation: 0.73							
Null model						3	4048.86	174.28	
Trend model					176.28	4	3874.58		<0.001

Distribution: Negative binomial. The mean number of independent photographs per camera-year was 34.17, and the variance was 1282.57, resulting in a dispersion index (variance/mean) of 37.54, indicating strong overdispersion.

Table S4. Results of the Generalized Linear Mixed Model testing temporal trends in relative abundance index of South China sika deer from 2015 to 2024.

Category	Parameter	Estimate	SE	z	χ^2	df	AICc	ΔAICc	*P*
Fixed effects	Intercept	0.86	0.12	7.03					<0.001
	Year	0.17	0.01	14.05					<0.001
Random effect			Variance: 0.49; Standard deviation: 0.70						
Null model						3	2892.89	167.63	
Trend model				169.62		4	2725.26		<0.001

Distribution: Negative binomial. The mean relative abundance index per camera-year was 9.36, and the variance was 96.27, resulting in a dispersion index (variance/mean) of 10.28, indicating overdispersion.

Table S5. Results of one-way ANOVA testing interannual variation in Leaf Area Index in relation to South China sika deer in the Qingliangfeng Biosphere Reserve from 2015 to 2024.

**Term**	**Df**	**Sum Sq**	**Mean Sq**	**F value**	**Pr(>F)**
Year	9	33.660	3.740	99.512	< 2e-16
Residuals	1060	39.830	0.038		

Table S6. Results of one-way ANOVA testing interannual variation in Normalized Difference Vegetation Index in relation to South China sika deer in Qingliangfeng Biosphere Reserve from 2015 to 2024.

Term	Df	Sum Sq	Mean Sq	F value	Pr(>F)
Year	9	0.461	0.051	34.402	< 2e-16
Residuals	1060	1.578	0.001		

Figure S2. Interannual variation in annual mean Leaf Area Index and Normalized Difference Vegetation Index in the Qingliangfeng Biosphere Reserve from 2015 to 2024.

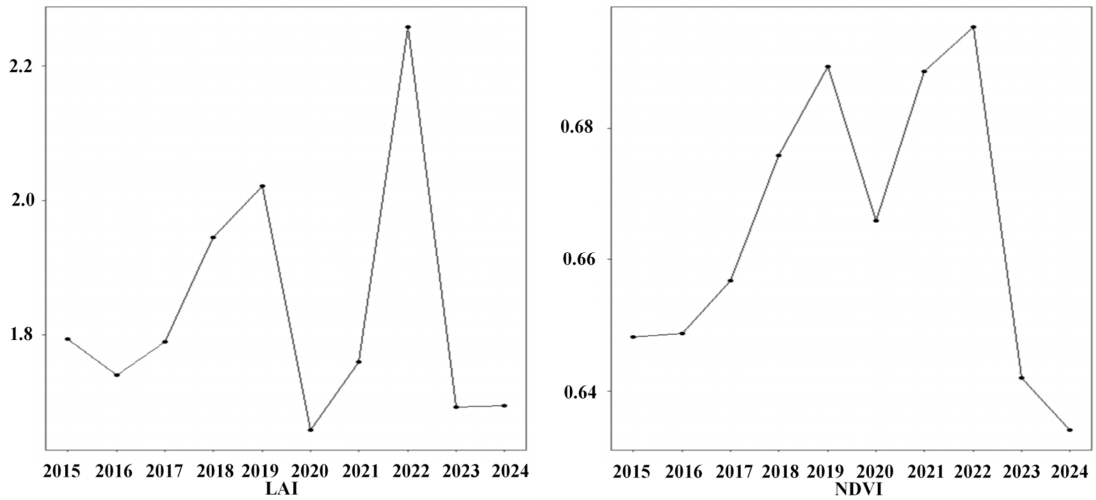

Table S7. Annual mean Normalized Difference Vegetation Index and Leaf Area Index in the Qingliangfeng Biosphere Reserve from 2015 to 2024.

Year	NDVI	LAI
2015	0.648	1.793
2016	0.649	1.740

Year	NDVI	LAI
2017	0.657	1.789
2018	0.676	1.945
2019	0.689	2.021
2020	0.666	1.659
2021	0.689	1.760
2022	0.695	2.257
2023	0.642	1.692
2024	0.634	1.695

Table S8. Result of segmented regression analysis of temporal changes in annual mean Normalized Difference Vegetation Index in the Qingliangfeng Biosphere Reserve from 2015 to 2024.

Parameter	Estimate	SE	t value	*P*	95% CI
Breakpoint year	2021.822	0.430	–	–	–
Slope before breakpoint	0.007	0.002	2.884	0.0280	[0.001, 0.013]
Slope after breakpoint	−0.031	0.001	−3.510	<0.010	[−0.052, −0.009]
Adjusted R^2	0.679	–	–	–	–
Residual SE	0.012	–	–	–	–

Table S9. Result of segmented regression analysis of temporal changes in annual mean Leaf Area Index in the Qingliangfeng Biosphere Reserve from 2015 to 2024.

Parameter	Estimate	SE	t value	*P*	95% CI
Estimated breakpoint (year)	2018.731	3.710	–	–	–
Slope before breakpoint	0.050	0.097	0.522	0.624	[−0.188, 0.289]
Slope after breakpoint	−0.030	0.052	−0.572	0.097	[−0.157, 0.098]

Parameter	Estimate	SE	t value	*P*	95% CI
Adjusted R^2	−0.356	–	–	–	–
Residual SE	0.218	–	–	–	–

Figure S3. Spatial patterns of multi-year mean Leaf Area Index and Normalized Difference Vegetation Index, and spatial distribution of their interannual trends in the Qingliangfeng Biosphere Reserve. (a) Spatial distribution of multi-year Leaf Area Index; (b) Spatial distribution of multi-year Normalized Difference Vegetation Index; (c) Spatial distribution of interannual trends in Leaf Area Index, represented by linear regression slopes; (d) Spatial distribution of interannual trends in Normalized Difference Vegetation Index, represented by linear regression slopes.

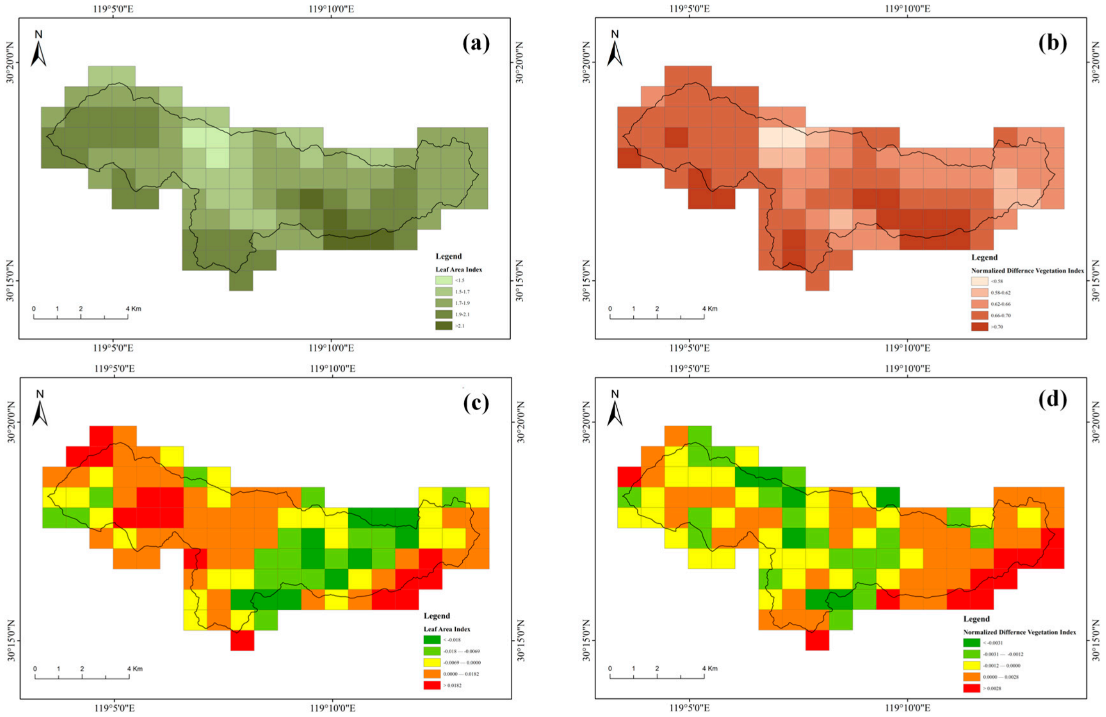

Table S10. AICc-based model ranking results in examining the relationships between the number of independent photographs of sika deer and explanatory variables from 2015 to 2024. Candidate models were derived from the global model including distance to road, distance to settlement, distance to water, elevation, slope, year, and Normalized Difference Vegetation Index.

Model	Intercept	Road	Settlement	Water	Elevation	NDVI	Slope	Year	df	logLik	AICc	delta	weight
1	2.185	0.250	-0.421	0.207	0.231			0.177	8	-1720.627	3457.612	0.000	0.324
2	2.186	0.259	-0.430	0.213	0.232		0.031	0.176	9	-1720.532	3459.511	1.899	0.125
3	2.189	0.245	-0.416	0.209	0.226	-0.019		0.176	9	-1720.554	3459.556	1.945	0.123
4	2.188	0.269	-0.391		0.341			0.178	7	-1723.025	3460.328	2.716	0.083
5	2.179	0.333	-0.479	0.334				0.176	7	-1723.470	3461.218	3.606	0.053
6	2.171		-0.295	0.228	0.304			0.177	7	-1723.522	3461.321	3.709	0.051
7	2.191	0.254	-0.425	0.216	0.225	-0.023	0.036	0.176	10	-1720.430	3461.408	3.797	0.049
8	2.189	0.266	-0.389		0.339	-0.008		0.177	8	-1723.012	3462.381	4.769	0.030
9	2.188	0.271	-0.393		0.342		0.007	0.177	8	-1723.020	3462.398	4.786	0.030
10	2.187	0.321	-0.467	0.332		-0.033		0.174	8	-1723.264	3462.885	5.274	0.023
11	2.178		-0.291	0.230	0.293	-0.030		0.176	8	-1723.349	3463.056	5.444	0.021
12	2.180	0.343	-0.487	0.340			0.030	0.175	8	-1723.391	3463.140	5.528	0.020
13	2.171		-0.295	0.226	0.304		-0.007	0.177	8	-1723.518	3463.393	5.781	0.018
14	2.190	0.269	-0.391		0.340	-0.009	0.009	0.177	9	-1723.004	3464.457	6.845	0.011
15	2.174		-0.253		0.432			0.178	6	-1726.190	3464.587	6.976	0.010
16	2.189	0.331	-0.476	0.339		-0.037	0.037	0.174	9	-1723.141	3464.731	7.119	0.009
17	2.178		-0.291	0.230	0.293	-0.030	0.000	0.176	9	-1723.349	3465.146	7.534	0.007
18	2.173		-0.250		0.425		-0.033	0.178	7	-1726.088	3466.453	8.842	0.004

19	2.178		-0.250		0.426	-0.019		0.177	7	-1726.120	3466.518	8.906	0.004
20	2.177		-0.248		0.420	-0.016	-0.030	0.177	8	-1726.039	3468.435	10.823	0.001
21	2.154		-0.319	0.424				0.175	6	-1728.409	3469.025	11.414	0.001
22	2.168		-0.311	0.415		-0.051		0.174	7	-1727.926	3470.130	12.518	0.001
23	2.154		-0.316	0.417			-0.027	0.176	7	-1728.353	3470.982	13.371	0.000
24	2.168		-0.309	0.412		-0.049	-0.014	0.174	8	-1727.910	3472.178	14.567	0.000
25	2.176				0.412			0.178	5	-1731.092	3472.331	14.719	0.000
26	2.173			0.146	0.327			0.177	6	-1730.111	3472.429	14.817	0.000
27	2.180	0.468	-0.474					0.176	6	-1730.627	3473.461	15.849	0.000
28	2.175				0.401		-0.055	0.178	6	-1730.849	3473.906	16.294	0.000
29	2.181			0.150	0.315	-0.032		0.176	7	-1729.925	3474.126	16.515	0.000
30	2.182				0.404	-0.025		0.177	6	-1730.973	3474.154	16.543	0.000
31	2.173			0.139	0.323		-0.041	0.178	7	-1729.977	3474.232	16.620	0.000
32	2.177	0.010			0.408			0.178	6	-1731.086	3474.379	16.767	0.000
33	2.172	-0.017		0.150	0.332			0.177	7	-1730.095	3474.467	16.855	0.000
34	2.186	0.458	-0.466			-0.024		0.175	7	-1730.519	3475.315	17.703	0.000
35	2.180	0.459	-0.468				-0.024	0.176	7	-1730.584	3475.446	17.834	0.000
36	2.180				0.395	-0.021	-0.050	0.177	7	-1730.771	3475.820	18.208	0.000
37	2.176	0.002			0.400		-0.055	0.178	7	-1730.849	3475.975	18.363	0.000
38	2.180			0.144	0.312	-0.028	-0.034	0.176	8	-1729.833	3476.023	18.411	0.000
39	2.180	-0.021		0.155	0.320	-0.033		0.176	8	-1729.901	3476.158	18.547	0.000
40	2.182	0.008			0.400	-0.025		0.177	7	-1730.970	3476.217	18.605	0.000
41	2.172	-0.023		0.144	0.329		-0.043	0.178	8	-1729.950	3476.257	18.645	0.000
42	2.185	0.451	-0.461			-0.023	-0.019	0.175	8	-1730.491	3477.339	19.727	0.000
43	2.180	0.001			0.394	-0.021	-0.050	0.177	8	-1730.771	3477.900	20.288	0.000
44	2.178	-0.025		0.149	0.318	-0.029	-0.036	0.176	9	-1729.798	3478.045	20.433	0.000

45	2.154			0.353				0.176	5	-1734.878	3479.904	22.293	0.000
46	2.168			0.345		-0.052		0.174	6	-1734.390	3480.988	23.377	0.000
47	2.154			0.337			-0.066	0.176	6	-1734.588	3481.383	23.771	0.000
48	2.158	0.057		0.332				0.176	6	-1734.725	3481.658	24.047	0.000
49	2.167			0.333		-0.047	-0.052	0.175	7	-1734.201	3482.679	25.068	0.000
50	2.171	0.047		0.328		-0.050		0.174	7	-1734.281	3482.839	25.227	0.000
51	2.158	0.048		0.320			-0.060	0.176	7	-1734.481	3483.240	25.628	0.000
52	2.170	0.041		0.319		-0.045	-0.048	0.175	8	-1734.120	3484.598	26.986	0.000
53	2.140		-0.228					0.176	5	-1739.074	3488.296	30.684	0.000
54	2.143		-0.218				-0.124	0.177	6	-1738.087	3488.382	30.770	0.000
55	2.153		-0.221			-0.047		0.174	6	-1738.686	3489.580	31.968	0.000
56	2.153		-0.214			-0.037	-0.114	0.175	7	-1737.849	3489.975	32.363	0.000
57	2.161	0.194						0.176	5	-1740.007	3490.163	32.551	0.000
58	2.161	0.169					-0.109	0.177	6	-1739.298	3490.804	33.192	0.000
59	2.146						-0.140	0.177	5	-1740.612	3491.372	33.760	0.000
60	2.172	0.184				-0.042		0.174	6	-1739.694	3491.595	33.983	0.000
61	2.143							0.176	4	-1741.783	3491.665	34.053	0.000
62	2.170	0.164				-0.034	-0.099	0.175	7	-1739.092	3492.462	34.850	0.000
63	2.157					-0.049		0.174	5	-1741.358	3492.864	35.252	0.000
64	2.156					-0.038	-0.128	0.175	6	-1740.358	3492.923	35.311	0.000
65	3.325	0.279	-0.448	0.344		-0.190	0.120		8	-1802.050	3620.457	162.846	0.000
66	3.328	0.249	-0.420	0.321		-0.178			7	-1803.340	3620.957	163.345	0.000
67	3.329	0.243	-0.423	0.284	0.110	-0.179	0.119		9	-1801.411	3621.270	163.659	0.000
68	3.332	0.213	-0.394	0.260	0.113	-0.167			8	-1802.699	3621.755	164.144	0.000
69	3.323		-0.283	0.277	0.172	-0.179			7	-1804.606	3623.489	165.877	0.000
70	3.320		-0.292	0.296	0.177	-0.189	0.087		8	-1803.933	3624.222	166.611	0.000

71	3.313		-0.294	0.384		-0.198		6	-1806.124	3624.456	166.844	0.000
72	3.310		-0.302	0.404		-0.209	0.081	7	-1805.571	3625.420	167.808	0.000
73	3.341	0.238	-0.356		0.256	-0.154		7	-1806.034	3626.345	168.733	0.000
74	3.339	0.262	-0.375		0.264	-0.161	0.086	8	-1805.413	3627.183	169.571	0.000
75	3.341	0.255	-0.435	0.239	0.170			7	-1807.146	3628.569	170.958	0.000
76	3.331		-0.230		0.334	-0.167		6	-1808.187	3628.581	170.969	0.000
77	3.334	0.316	-0.478	0.331				6	-1808.483	3629.173	171.561	0.000
78	3.339	0.282	-0.459	0.255	0.171		0.091	8	-1806.454	3629.266	171.654	0.000
79	3.332	0.343	-0.502	0.349			0.090	7	-1807.834	3629.945	172.333	0.000
80	3.329		-0.234		0.343	-0.172	0.051	7	-1807.977	3630.232	172.620	0.000
81	3.329		-0.304	0.258	0.247			6	-1809.663	3631.533	173.921	0.000
82	3.350	0.276	-0.398		0.298			6	-1809.762	3631.732	174.120	0.000
83	3.333	0.376	-0.411			-0.176		6	-1809.997	3632.202	174.591	0.000
84	3.327			0.196	0.197	-0.185		6	-1810.180	3632.568	174.956	0.000
85	3.348	0.295	-0.413		0.306		0.063	7	-1809.443	3633.163	175.551	0.000
86	3.327		-0.310	0.269	0.252		0.052	7	-1809.448	3633.174	175.562	0.000
87	3.331	0.399	-0.426			-0.182	0.067	7	-1809.669	3633.616	176.005	0.000
88	3.333				0.314	-0.176		5	-1811.814	3633.775	176.163	0.000
89	3.315			0.316		-0.205		5	-1811.858	3633.865	176.253	0.000
90	3.325			0.206	0.201	-0.192	0.058	7	-1809.932	3634.142	176.530	0.000
91	3.325	-0.039		0.205	0.206	-0.187		7	-1810.103	3634.484	176.872	0.000
92	3.313		-0.324	0.416				5	-1812.474	3635.096	177.484	0.000
93	3.338		-0.254		0.393			5	-1812.491	3635.130	177.518	0.000
94	3.313			0.326		-0.210	0.049	6	-1811.688	3635.584	177.972	0.000
95	3.332				0.320	-0.180	0.035	6	-1811.725	3635.658	178.046	0.000
96	3.333	0.000			0.314	-0.176		6	-1811.814	3635.835	178.223	0.000

97	3.315	0.002		0.315		-0.205		6	-1811.858	3635.924	178.312	0.000
98	3.323	-0.033		0.213	0.208	-0.193	0.055	8	-1809.878	3636.113	178.502	0.000
99	3.311		-0.328	0.426			0.037	6	-1812.378	3636.962	179.351	0.000
100	3.337		-0.256		0.398		0.021	6	-1812.457	3637.121	179.509	0.000
101	3.313	0.008		0.324		-0.210	0.050	7	-1811.684	3637.646	180.034	0.000
102	3.332	0.005			0.318	-0.180	0.036	7	-1811.724	3637.725	180.113	0.000
103	3.340	0.447	-0.469					5	-1814.817	3639.782	182.171	0.000
104	3.335			0.171	0.276			5	-1815.415	3640.977	183.365	0.000
105	3.307		-0.202			-0.205		5	-1815.492	3641.133	183.521	0.000
106	3.341				0.375			4	-1816.542	3641.181	183.570	0.000
107	3.339	0.461	-0.479				0.037	6	-1814.726	3641.660	184.048	0.000
108	3.334			0.174	0.278		0.019	6	-1815.391	3642.989	185.377	0.000
109	3.334	-0.020		0.176	0.281			6	-1815.397	3643.002	185.390	0.000
110	3.308		-0.202			-0.204	-0.010	6	-1815.486	3643.180	185.569	0.000
111	3.341	0.013			0.370			5	-1816.534	3643.215	185.604	0.000
112	3.340				0.375		0.002	5	-1816.541	3643.231	185.619	0.000
113	3.310					-0.209		4	-1817.803	3643.704	186.092	0.000
114	3.321	0.133				-0.201		5	-1816.912	3643.971	186.359	0.000
115	3.316			0.344				4	-1818.357	3644.813	187.201	0.000
116	3.333	-0.017		0.178	0.282		0.017	7	-1815.377	3645.031	187.420	0.000
117	3.341	0.014			0.370		0.003	6	-1816.533	3645.274	187.662	0.000
118	3.311					-0.207	-0.020	5	-1817.777	3645.703	188.091	0.000
119	3.321	0.133				-0.201	0.002	6	-1816.911	3646.030	188.418	0.000
120	3.319	0.042		0.328				5	-1818.283	3646.714	189.103	0.000
121	3.316			0.344			0.000	5	-1818.357	3646.862	189.251	0.000
122	3.319	0.042		0.329			0.005	6	-1818.282	3648.771	191.160	0.000

123	3.307		-0.227		4	-1821.841	3651.780	194.168	0.000
124	3.310		-0.223	-0.059	5	-1821.636	3653.419	195.807	0.000
125	3.326	0.177			4	-1822.951	3654.000	196.388	0.000
126	3.310				3	-1824.307	3654.672	197.060	0.000
127	3.327	0.167		-0.043	5	-1822.852	3655.851	198.239	0.000
128	3.314			-0.073	4	-1824.019	3656.136	198.524	0.000

Table S11. Likelihood ratio tests comparing nested candidate models with the best model (Independent Photographs ~ Distance to road + Distance to settlement + Distance to water + Elevation + Year).

Models	χ^2	*P*
Model (Distance to road, Distance to settlement, Distance to water, Elevation, Slope, Year) VS Best model	0.2069	0.6492
Model (Distance to road, Distance to settlement, Distance to water, Elevation, NDVI, Year) VS Best model	0.1512	0.6974
Model (Distance to road, Distance to settlement, Elevation, Year) VS Best model	4.6104	0.0318
Model (Distance to road, Distance to settlement, Distance to water, Year) VS Best model	5.7341	0.0166
Model (Distance to settlement, Distance to water, Elevation, Year) VS Best model	5.7417	0.0166
Model (Distance to road, Distance to settlement, Distance to water, Elevation, NDVI, Slope, Year) VS Best model	0.4191	0.8109

Table S12. AICc-based model ranking results in examining the relationships between the number of independent photographs of sika deer and explanatory variables from 2015 to 2024. Candidate models were derived from the global model including distance to road, distance to settlement, distance to water, elevation, slope, year, and Leaf Area Index.

Model	Intercept	Road	Settlement	Water	Elevation	LAI	Slope	Year	df	logLik	AICc	delta	weight
1	2.185	0.250	-0.421	0.207	0.231			0.177	8	-1720.627	3457.612	0.000	0.297
2	2.186	0.241	-0.413	0.212	0.220	-0.035		0.177	9	-1720.278	3459.003	1.392	0.148
3	2.186	0.259	-0.430	0.213	0.232		0.031	0.176	9	-1720.532	3459.511	1.899	0.115
4	2.188	0.269	-0.391		0.341			0.178	7	-1723.025	3460.328	2.716	0.076
5	2.188	0.251	-0.422	0.219	0.220	-0.036	0.033	0.176	10	-1720.166	3460.880	3.268	0.058
6	2.179	0.333	-0.479	0.334				0.176	7	-1723.470	3461.218	3.606	0.049
7	2.171		-0.295	0.228	0.304			0.177	7	-1723.522	3461.321	3.709	0.046
8	2.189	0.262	-0.384		0.335	-0.027		0.177	8	-1722.815	3461.988	4.376	0.033
9	2.183	0.316	-0.465	0.332		-0.045		0.175	8	-1722.877	3462.112	4.501	0.031
10	2.174		-0.291	0.232	0.288	-0.042		0.177	8	-1723.004	3462.366	4.754	0.028
11	2.188	0.271	-0.393		0.342		0.007	0.177	8	-1723.020	3462.398	4.786	0.027
12	2.180	0.343	-0.487	0.340			0.030	0.175	8	-1723.391	3463.140	5.528	0.019
13	2.171		-0.295	0.226	0.304		-0.007	0.177	8	-1723.518	3463.393	5.781	0.016
14	2.184	0.326	-0.474	0.339		-0.046	0.033	0.175	9	-1722.778	3464.004	6.393	0.012
15	2.189	0.264	-0.387		0.336	-0.027	0.009	0.177	9	-1722.808	3464.065	6.453	0.012
16	2.174		-0.291	0.232	0.287	-0.042	-0.002	0.177	9	-1723.004	3464.456	6.844	0.010
17	2.174		-0.253		0.432			0.178	6	-1726.190	3464.587	6.976	0.009
18	2.176		-0.249		0.421	-0.035		0.177	7	-1725.843	3465.963	8.351	0.005

19	2.173		-0.250		0.425		-0.033	0.178	7	-1726.088	3466.453	8.842	0.004
20	2.175		-0.246		0.414	-0.034	-0.030	0.178	8	-1725.757	3467.870	10.259	0.002
21	2.154		-0.319	0.424				0.175	6	-1728.409	3469.025	11.414	0.001
22	2.160		-0.312	0.416		-0.058		0.175	7	-1727.451	3469.180	11.568	0.001
23	2.154		-0.316	0.417			-0.027	0.176	7	-1728.353	3470.982	13.371	0.000
24	2.160		-0.310	0.410		-0.057	-0.019	0.175	8	-1727.421	3471.199	13.587	0.000
25	2.176				0.412			0.178	5	-1731.092	3472.331	14.719	0.000
26	2.173			0.146	0.327			0.177	6	-1730.111	3472.429	14.817	0.000
27	2.180	0.468	-0.474					0.176	6	-1730.627	3473.461	15.849	0.000
28	2.176			0.152	0.310	-0.042		0.177	7	-1729.596	3473.470	15.858	0.000
29	2.179				0.400	-0.038		0.177	6	-1730.677	3473.561	15.950	0.000
30	2.175				0.401		-0.055	0.178	6	-1730.849	3473.906	16.294	0.000
31	2.173			0.139	0.323		-0.041	0.178	7	-1729.977	3474.232	16.620	0.000
32	2.177	0.010			0.408			0.178	6	-1731.086	3474.379	16.767	0.000
33	2.172	-0.017		0.150	0.332			0.177	7	-1730.095	3474.467	16.855	0.000
34	2.183	0.452	-0.462			-0.040		0.176	7	-1730.163	3474.604	16.992	0.000
35	2.178				0.389	-0.037	-0.052	0.178	7	-1730.461	3475.200	17.588	0.000
36	2.176			0.146	0.307	-0.041	-0.036	0.177	8	-1729.490	3475.337	17.725	0.000
37	2.180	0.459	-0.468				-0.024	0.176	7	-1730.584	3475.446	17.834	0.000
38	2.175	-0.023		0.157	0.316	-0.043		0.177	8	-1729.568	3475.494	17.882	0.000
39	2.179	0.007			0.397	-0.038		0.177	7	-1730.674	3475.626	18.014	0.000
40	2.176	0.002			0.400		-0.055	0.178	7	-1730.849	3475.975	18.363	0.000
41	2.172	-0.023		0.144	0.329		-0.043	0.178	8	-1729.950	3476.257	18.645	0.000
42	2.183	0.444	-0.457			-0.040	-0.021	0.176	8	-1730.130	3476.617	19.005	0.000
43	2.178	-0.001			0.390	-0.037	-0.052	0.178	8	-1730.461	3477.279	19.668	0.000
44	2.174	-0.028		0.151	0.313	-0.042	-0.039	0.177	9	-1729.449	3477.345	19.734	0.000

45	2.154			0.353				0.176	5	-1734.878	3479.904	22.293	0.000
46	2.160			0.346		-0.057		0.175	6	-1733.955	3480.118	22.506	0.000
47	2.154			0.337			-0.066	0.176	6	-1734.588	3481.383	23.771	0.000
48	2.158	0.057		0.332				0.176	6	-1734.725	3481.658	24.047	0.000
49	2.160			0.332		-0.055	-0.057	0.176	7	-1733.724	3481.725	24.113	0.000
50	2.163	0.045		0.329		-0.055		0.175	7	-1733.854	3481.985	24.373	0.000
51	2.158	0.048		0.320			-0.060	0.176	7	-1734.481	3483.240	25.628	0.000
52	2.163	0.038		0.319		-0.054	-0.053	0.176	8	-1733.655	3483.667	26.055	0.000
53	2.140		-0.228					0.176	5	-1739.074	3488.296	30.684	0.000
54	2.143		-0.218				-0.124	0.177	6	-1738.087	3488.382	30.770	0.000
55	2.147		-0.222			-0.055		0.175	6	-1738.209	3488.626	31.015	0.000
56	2.149		-0.214			-0.052	-0.116	0.176	7	-1737.318	3488.913	31.302	0.000
57	2.161	0.194						0.176	5	-1740.007	3490.163	32.551	0.000
58	2.166	0.182				-0.051		0.176	6	-1739.274	3490.756	33.144	0.000
59	2.161	0.169					-0.109	0.177	6	-1739.298	3490.804	33.192	0.000
60	2.146						-0.140	0.177	5	-1740.612	3491.372	33.760	0.000
61	2.166	0.160				-0.049	-0.102	0.176	7	-1738.626	3491.529	33.918	0.000
62	2.143							0.176	4	-1741.783	3491.665	34.053	0.000
63	2.152					-0.052	-0.131	0.176	6	-1739.838	3491.884	34.272	0.000
64	2.150					-0.056		0.175	5	-1740.904	3491.955	34.344	0.000
65	3.334	0.287	-0.458	0.328		-0.081			7	-1807.013	3628.303	170.692	0.000
66	3.340	0.238	-0.423	0.249	0.146	-0.071			8	-1806.022	3628.401	170.790	0.000
67	3.341	0.255	-0.435	0.239	0.170				7	-1807.146	3628.569	170.958	0.000
68	3.331	0.314	-0.483	0.346		-0.084	0.097		8	-1806.244	3628.845	171.233	0.000
69	3.337	0.266	-0.448	0.267	0.146	-0.075	0.096		9	-1805.229	3628.905	171.293	0.000
70	3.334	0.316	-0.478	0.331					6	-1808.483	3629.173	171.561	0.000

71	3.339	0.282	-0.459	0.255	0.171		0.091	8	-1806.454	3629.266	171.654	0.000
72	3.332	0.343	-0.502	0.349			0.090	7	-1807.834	3629.945	172.333	0.000
73	3.329		-0.300	0.267	0.214	-0.080		7	-1808.262	3630.801	173.190	0.000
74	3.329		-0.304	0.258	0.247			6	-1809.663	3631.533	173.921	0.000
75	3.350	0.276	-0.398		0.298			6	-1809.762	3631.732	174.120	0.000
76	3.348	0.262	-0.386		0.282	-0.063		7	-1808.891	3632.060	174.448	0.000
77	3.326		-0.307	0.280	0.219	-0.082	0.060	8	-1807.962	3632.282	174.670	0.000
78	3.315		-0.316	0.402		-0.095		6	-1810.428	3633.063	175.451	0.000
79	3.348	0.295	-0.413		0.306		0.063	7	-1809.443	3633.163	175.551	0.000
80	3.327		-0.310	0.269	0.252		0.052	7	-1809.448	3633.174	175.562	0.000
81	3.347	0.282	-0.401		0.289	-0.065	0.067	8	-1808.530	3633.418	175.807	0.000
82	3.313		-0.322	0.415		-0.098	0.049	7	-1810.242	3634.761	177.150	0.000
83	3.337		-0.249		0.369	-0.072		6	-1811.367	3634.941	177.329	0.000
84	3.313		-0.324	0.416				5	-1812.474	3635.096	177.484	0.000
85	3.338		-0.254		0.393			5	-1812.491	3635.130	177.518	0.000
86	3.336		-0.251		0.375	-0.073	0.028	7	-1811.308	3636.893	179.281	0.000
87	3.311		-0.328	0.426			0.037	6	-1812.378	3636.962	179.351	0.000
88	3.337		-0.256		0.398		0.021	6	-1812.457	3637.121	179.509	0.000
89	3.340	0.417	-0.449			-0.080		6	-1813.414	3639.036	181.424	0.000
90	3.340	0.447	-0.469					5	-1814.817	3639.782	182.171	0.000
91	3.334			0.181	0.243	-0.079		6	-1814.039	3640.286	182.674	0.000
92	3.340				0.350	-0.075		5	-1815.327	3640.802	183.190	0.000
93	3.338	0.433	-0.460			-0.081	0.043	7	-1813.285	3640.846	183.235	0.000
94	3.335			0.171	0.276			5	-1815.415	3640.977	183.365	0.000
95	3.341				0.375			4	-1816.542	3641.181	183.570	0.000
96	3.339	0.461	-0.479				0.037	6	-1814.726	3641.660	184.048	0.000

97	3.333			0.186	0.246	-0.080	0.028	7	-1813.986	3642.250	184.638	0.000
98	3.332	-0.030		0.188	0.251	-0.080		7	-1813.998	3642.274	184.662	0.000
99	3.339				0.352	-0.075	0.009	6	-1815.322	3642.851	185.240	0.000
100	3.340	0.006			0.348	-0.075		6	-1815.325	3642.858	185.247	0.000
101	3.318			0.331		-0.094		5	-1816.378	3642.903	185.292	0.000
102	3.334			0.174	0.278		0.019	6	-1815.391	3642.989	185.377	0.000
103	3.334	-0.020		0.176	0.281			6	-1815.397	3643.002	185.390	0.000
104	3.341	0.013			0.370			5	-1816.534	3643.215	185.604	0.000
105	3.340				0.375		0.002	5	-1816.541	3643.231	185.619	0.000
106	3.331	-0.027		0.191	0.252	-0.081	0.026	8	-1813.954	3644.265	186.653	0.000
107	3.316			0.344				4	-1818.357	3644.813	187.201	0.000
108	3.340	0.007			0.349	-0.075	0.009	7	-1815.320	3644.916	187.305	0.000
109	3.319	0.022		0.323		-0.093		6	-1816.356	3644.919	187.307	0.000
110	3.317			0.334		-0.095	0.013	6	-1816.366	3644.940	187.329	0.000
111	3.333	-0.017		0.178	0.282		0.017	7	-1815.377	3645.031	187.420	0.000
112	3.341	0.014			0.370		0.003	6	-1816.533	3645.274	187.662	0.000
113	3.319	0.042		0.328				5	-1818.283	3646.714	189.103	0.000
114	3.316			0.344			0.000	5	-1818.357	3646.862	189.251	0.000
115	3.319	0.024		0.325		-0.094	0.016	7	-1816.340	3646.958	189.346	0.000
116	3.319	0.042		0.329			0.005	6	-1818.282	3648.771	191.160	0.000
117	3.310		-0.222			-0.099		5	-1819.712	3649.572	191.960	0.000
118	3.312		-0.219			-0.097	-0.044	6	-1819.593	3651.393	193.782	0.000
119	3.307		-0.227					4	-1821.841	3651.780	194.168	0.000
120	3.326	0.156				-0.092		5	-1821.109	3652.366	194.754	0.000
121	3.313					-0.098		4	-1822.209	3652.516	194.904	0.000
122	3.310		-0.223				-0.059	5	-1821.636	3653.419	195.807	0.000

123	3.326	0.177			4	-1822.951	3654.000	196.388	0.000
124	3.316		-0.096	-0.057	5	-1822.022	3654.192	196.580	0.000
125	3.327	0.149	-0.091	-0.032	6	-1821.053	3654.313	196.702	0.000
126	3.310				3	-1824.307	3654.672	197.060	0.000
127	3.327	0.167		-0.043	5	-1822.852	3655.851	198.239	0.000
128	3.314			-0.073	4	-1824.019	3656.136	198.524	0.000

Table S13. Likelihood ratio tests for comparing nested candidate models with the best model (Independent Photographs ~ Distance to road + Distance to settlement + Distance to water + Elevation + Year).

Models	χ^2	P
Model (Distance to road, Distance to settlement, Distance to water, Elevation, LAI, Year) VS Best model	0.7440	0.3884
Model (Distance to road, Distance to settlement, Distance to water, Elevation, Slope, Year) VS Best model	0.2069	0.6492
Model (Distance to road, Distance to settlement, Elevation, Year) VS Best model)	4.6104	0.0318
Model (Distance to road, Distance to settlement, Distance to water, Elevation, LAI, Slope, Year) VS Best model	0.9862	0.6107
Model (Distance to road, Distance to settlement, Distance to water, Year) VS Best model	5.7341	0.0166
Model (Distance to settlement, Distance to water, Elevation, Year) VS Best model	5.7417	0.0166

Table S14. Results of year-specific candidate models with ΔAICc < 4 assessing the relationships between independent photograph counts of sika deer and explanatory variables (distance to road, distance to settlement, distance to water, elevation, slope, and Leaf Area Index) from 2015 to 2024.

Year	Models	k	AICc	ΔAICc
2015	independent photographs ~ distance to settlement + distance to water + LAI	3	320.856	0.000
2015	independent photographs ~ elevation + distance to settlement + distance to water + LAI	4	322.607	1.752
2015	independent photographs ~ distance to road + distance to settlement + distance to water + LAI	4	323.045	2.190
2015	independent photographs ~ slope + distance to settlement + distance to water + LAI	4	323.094	2.238
2015	independent photographs ~ elevation + distance to settlement + LAI	3	324.266	3.410
2015	independent photographs ~ distance to water + LAI	2	324.750	3.894
2016	independent photographs ~ elevation + LAI	2	296.178	0.000
2016	independent photographs ~ elevation + distance to water + LAI	3	298.165	1.987
2016	independent photographs ~ elevation + distance to settlement + LAI	3	298.218	2.040
2016	independent photographs ~ elevation + slope + LAI	3	298.286	2.108
2016	independent photographs ~ elevation + distance to road + LAI	3	298.510	2.332
2016	independent photographs ~ distance to water + LAI	2	298.842	2.664
2017	independent photographs ~ elevation	1	295.505	0.000
2017	independent photographs ~ elevation + slope	2	296.747	1.242
2017	independent photographs ~ elevation + LAI	2	296.824	1.318
2017	independent photographs ~ distance to water + LAI	2	297.357	1.852
2017	independent photographs ~ elevation + distance to settlement	2	297.409	1.904
2017	independent photographs ~ elevation + distance to water	2	297.641	2.136
2017	independent photographs ~ elevation + distance to road	2	297.751	2.246
2017	independent photographs ~ distance to water	1	297.795	2.289
2017	independent photographs ~ LAI	1	298.242	2.737
2017	independent photographs ~ elevation + slope + LAI	3	298.539	3.034

2017	independent photographs ~ elevation + distance to settlement + LAI	3	298.671	3.166
2017	independent photographs ~ distance to settlement + distance to water + LAI	3	298.745	3.239
2017	independent photographs ~ elevation + distance to water + LAI	3	298.803	3.298
2017	independent photographs ~ elevation + slope + distance to water	3	298.822	3.316
2017	independent photographs ~ elevation + slope + distance to settlement	3	299.007	3.501
2017	independent photographs ~ elevation + distance to road + LAI	3	299.020	3.515
2017	independent photographs ~ elevation + slope + distance to road	3	299.090	3.585
2017	independent photographs ~ slope + distance to water	2	299.102	3.597
2017	independent photographs ~ elevation + distance to road + distance to settlement	3	299.122	3.617
2017	independent photographs ~ distance to road + distance to water + LAI	3	299.159	3.654
2017	independent photographs ~ slope + distance to water + LAI	3	299.181	3.676
2017	independent photographs ~ elevation + distance to settlement + distance to water	3	299.504	3.999
2018	independent photographs ~ elevation + distance to road + distance to settlement	3	340.629	0.000
2018	independent photographs ~ elevation + distance to settlement	2	341.442	0.813
2018	independent photographs ~ elevation + distance to settlement + LAI	3	342.221	1.591
2018	independent photographs ~ elevation + distance to road + distance to settlement + LAI	4	342.728	2.098
2018	independent photographs ~ elevation + distance to road + distance to settlement + distance to water	4	342.990	2.361
2018	independent photographs ~ elevation + slope + distance to road + distance to settlement	4	343.040	2.411
2018	independent photographs ~ elevation + distance to settlement + distance to water	3	343.626	2.997
2018	independent photographs ~ elevation + slope + distance to settlement	3	343.722	3.093
2018	independent photographs ~ elevation	1	344.093	3.464
2018	independent photographs ~ elevation + LAI	2	344.353	3.723

2018	independent photographs ~ elevation + distance to settlement + distance to water + LAI	4	344.449	3.820
2018	independent photographs ~ elevation + slope + distance to settlement + LAI	4	344.616	3.987
2019	independent photographs ~ elevation + LAI	2	298.777	0.000
2019	independent photographs ~ elevation + slope + LAI	3	299.735	0.957
2019	independent photographs ~ elevation + distance to road + distance to water	3	300.180	1.403
2019	independent photographs ~ elevation + distance to road + LAI	3	300.623	1.846
2019	independent photographs ~ elevation + distance to water + LAI	3	300.718	1.940
2019	independent photographs ~ elevation + distance to settlement + LAI	3	301.143	2.366
2019	independent photographs ~ elevation + slope + distance to water + LAI	4	301.308	2.531
2019	independent photographs ~ elevation + slope + distance to road + distance to water	4	301.559	2.781
2019	independent photographs ~ elevation + distance to road + distance to settlement + distance to water	4	301.898	3.120
2019	independent photographs ~ elevation + distance to water	2	301.929	3.152
2019	independent photographs ~ elevation + distance to road + distance to water + LAI	4	301.947	3.170
2019	independent photographs ~ elevation + distance to road + distance to settlement	3	302.001	3.224
2019	independent photographs ~ elevation + slope + distance to road + LAI	4	302.034	3.257
2019	independent photographs ~ elevation + slope + distance to settlement + LAI	4	302.190	3.413
2019	independent photographs ~ elevation + distance to road + distance to settlement + LAI	4	302.274	3.497
2019	independent photographs ~ elevation + distance to road	2	302.679	3.902
2019	independent photographs ~ elevation + slope + distance to water	3	302.755	3.978
2020	independent photographs ~ elevation + distance to settlement + LAI	3	262.639	0.000
2020	independent photographs ~ elevation + distance to road + distance to settlement	3	263.173	0.534
2020	independent photographs ~ elevation + distance to road + distance to settlement + LAI	4	263.617	0.978

2020	independent photographs ~ elevation + distance to settlement	2	263.730	1.091
2020	independent photographs ~ elevation + distance to settlement + distance to water + LAI	4	265.138	2.499
2020	independent photographs ~ elevation + slope + distance to settlement + LAI	4	265.261	2.622
2020	independent photographs ~ elevation + distance to road + distance to settlement + distance to water	4	265.276	2.637
2020	independent photographs ~ elevation + slope + distance to road + distance to settlement	4	265.377	2.738
2020	independent photographs ~ elevation + distance to settlement + distance to water	3	265.758	3.119
2020	independent photographs ~ elevation + LAI	2	265.858	3.219
2020	independent photographs ~ elevation + slope + distance to settlement	3	266.134	3.495
2020	independent photographs ~ elevation + slope + distance to road + distance to settlement + LAI	5	266.260	3.622
2020	independent photographs ~ elevation + distance to road + distance to settlement + distance to water + LAI	5	266.435	3.797
2020	independent photographs ~ elevation + distance to road + LAI	3	266.616	3.977
2021	independent photographs ~ elevation + distance to settlement	2	340.380	0.000
2021	independent photographs ~ elevation + distance to settlement + distance to water	3	341.238	0.858
2021	independent photographs ~ elevation + distance to road + distance to settlement	3	341.451	1.071
2021	independent photographs ~ elevation + distance to road + distance to settlement + distance to water	4	342.438	2.058
2021	independent photographs ~ elevation + slope + distance to settlement	3	342.629	2.249
2021	independent photographs ~ elevation + distance to settlement + LAI	3	342.722	2.341
2021	independent photographs ~ elevation + distance to settlement + distance to water + LAI	4	343.401	3.021
2021	independent photographs ~ elevation + slope + distance to settlement + distance to water	4	343.714	3.333
2021	independent photographs ~ elevation + distance to road + distance to settlement + LAI	4	343.722	3.341
2021	independent photographs ~ elevation + slope + distance to road + distance to settlement	4	343.906	3.526

2022	independent photographs ~ distance to road + distance to settlement + distance to water	3	309.576	0.000
2022	independent photographs ~ elevation + distance to road + distance to settlement + distance to water	4	310.136	0.559
2022	independent photographs ~ slope + distance to road + distance to settlement + distance to water	4	310.247	0.671
2022	independent photographs ~ distance to road + distance to settlement + distance to water + LAI	4	310.661	1.085
2022	independent photographs ~ elevation + distance to road + distance to settlement	3	311.153	1.577
2022	independent photographs ~ slope + distance to road + distance to settlement + distance to water + LAI	5	311.156	1.580
2022	independent photographs ~ elevation + slope + distance to road + distance to settlement + distance to water	5	311.230	1.654
2022	independent photographs ~ elevation + distance to road + distance to settlement + distance to water + LAI	5	312.409	2.833
2022	independent photographs ~ elevation + slope + distance to road + distance to settlement	4	312.703	3.127
2022	independent photographs ~ elevation + slope + distance to road + distance to settlement + distance to water + LAI	6	313.448	3.872
2023	independent photographs ~ slope + distance to settlement + distance to water	3	543.189	0.000
2023	independent photographs ~ distance to settlement + distance to water	2	543.298	0.109
2023	independent photographs ~ distance to road + distance to settlement + distance to water	3	543.973	0.783
2023	independent photographs ~ elevation + distance to road + distance to settlement + distance to water	4	544.253	1.063
2023	independent photographs ~ elevation + slope + distance to settlement + distance to water	4	544.526	1.337
2023	independent photographs ~ elevation + slope + distance to road + distance to settlement + distance to water	5	544.581	1.392
2023	independent photographs ~ slope + distance to road + distance to settlement + distance to water	4	544.613	1.424
2023	independent photographs ~ elevation + distance to settlement + distance to water + LAI	4	544.914	1.725
2023	independent photographs ~ elevation + distance to settlement + distance to water	3	545.025	1.836
2023	independent photographs ~ slope + distance to water	2	545.034	1.845
2023	independent photographs ~ distance to settlement + distance to water + LAI	3	545.470	2.281

2023	independent photographs ~ slope + distance to settlement + distance to water + LAI	4	545.480	2.291
2023	independent photographs ~ elevation + slope + distance to settlement + distance to water + LAI	5	545.543	2.353
2023	independent photographs ~ distance to road + distance to settlement + distance to water + LAI	4	545.833	2.644
2023	independent photographs ~ slope + distance to road + distance to settlement + distance to water + LAI	5	546.069	2.879
2023	independent photographs ~ elevation + distance to road + distance to settlement + distance to water + LAI	5	546.087	2.898
2023	independent photographs ~ distance to water	1	546.790	3.601
2023	independent photographs ~ elevation + slope + distance to road + distance to settlement + distance to water + LAI	6	546.871	3.682
2023	independent photographs ~ elevation + slope + distance to water	3	547.055	3.866
2023	independent photographs ~ elevation + slope + distance to water + LAI	4	547.089	3.900
2023	independent photographs ~ slope + distance to water + LAI	3	547.097	3.907
2024	independent photographs ~ distance to settlement + distance to water	2	492.867	0.000
2024	independent photographs ~ distance to water	1	492.960	0.093
2024	independent photographs ~ slope + distance to settlement + distance to water	3	493.650	0.782
2024	independent photographs ~ distance to road + distance to settlement + distance to water	3	494.545	1.677
2024	independent photographs ~ slope + distance to water	2	494.653	1.785
2024	independent photographs ~ slope + distance to road + distance to settlement + distance to water	4	494.886	2.019
2024	independent photographs ~ elevation + distance to settlement + distance to water	3	494.918	2.051
2024	independent photographs ~ distance to road + distance to water	2	495.117	2.250
2024	independent photographs ~ elevation + distance to water	2	495.119	2.252
2024	independent photographs ~ distance to water + LAI	2	495.126	2.259
2024	independent photographs ~ distance to settlement + distance to water + LAI	3	495.131	2.264
2024	independent photographs ~ elevation + slope + distance to settlement + distance to water	4	495.857	2.990
2024	independent photographs ~ slope + distance to settlement + distance to water + LAI	4	495.966	3.099

2024	independent photographs ~ elevation + distance to road + distance to settlement + distance to water	4	496.037	3.170
2024	independent photographs ~ elevation + slope + distance to road + distance to settlement + distance to water	5	496.353	3.486
2024	independent photographs ~ distance to road + distance to settlement + distance to water + LAI	4	496.591	3.724

Table S15. Results of year-specific candidate models with ΔAICc < 4 assessing the relationships between independent photograph counts of sika deer and explanatory variables (distance to road, distance to settlement, distance to water, elevation, slope, and Normalized Difference Vegetation Index) from 2015 to 2024.

Year	model	k	AICc	ΔAICc
2015	independent photographs ~ elevation + distance to settlement	2	327.123	0.000
2015	independent photographs ~ elevation + distance to road + distance to settlement	3	327.231	0.108
2015	independent photographs ~ elevation + distance to settlement + distance to water	3	328.946	1.823
2015	independent photographs ~ elevation + distance to road + distance to settlement + NDVI	4	329.019	1.896
2015	independent photographs ~ elevation + distance to road + distance to settlement + distance to water	4	329.105	1.982
2015	independent photographs ~ elevation + slope + distance to settlement	3	329.205	2.082
2015	independent photographs ~ elevation + distance to settlement + NDVI	3	329.278	2.156
2015	independent photographs ~ elevation + slope + distance to road + distance to settlement	4	329.289	2.166
2015	independent photographs ~ elevation + slope + distance to settlement + distance to water	4	330.929	3.806
2015	independent photographs ~ elevation + slope + distance to road + distance to settlement + distance to water	5	331.016	3.894
2016	independent photographs ~ elevation + distance to road + distance to settlement	3	307.650	0.000

2016	independent photographs ~ elevation + distance to settlement	2	308.050	0.400
2016	independent photographs ~ elevation + distance to road + distance to settlement + distance to water	4	309.821	2.170
2016	independent photographs ~ elevation + distance to settlement + NDVI	3	309.848	2.198
2016	independent photographs ~ elevation + distance to road + distance to settlement + NDVI	4	310.017	2.367
2016	independent photographs ~ elevation + slope + distance to road + distance to settlement	4	310.089	2.439
2016	independent photographs ~ elevation + distance to settlement + distance to water	3	310.191	2.540
2016	independent photographs ~ elevation + slope + distance to settlement	3	310.334	2.684
2017	independent photographs ~ elevation	1	295.505	0.000
2017	independent photographs ~ elevation + slope	2	296.747	1.242
2017	independent photographs ~ elevation + distance to settlement	2	297.409	1.904
2017	independent photographs ~ elevation + NDVI	2	297.430	1.925
2017	independent photographs ~ elevation + distance to water	2	297.641	2.136
2017	independent photographs ~ elevation + distance to road	2	297.751	2.246
2017	independent photographs ~ distance to water	1	297.795	2.289
2017	independent photographs ~ distance to water + NDVI	2	297.904	2.399
2017	independent photographs ~ elevation + slope + distance to water	3	298.822	3.316
2017	independent photographs ~ elevation + slope + NDVI	3	298.904	3.399
2017	independent photographs ~ elevation + slope + distance to settlement	3	299.007	3.501
2017	independent photographs ~ elevation + slope + distance to road	3	299.090	3.585
2017	independent photographs ~ slope + distance to water	2	299.102	3.597
2017	independent photographs ~ elevation + distance to road + distance to settlement	3	299.122	3.617
2017	independent photographs ~ elevation + distance to water + NDVI	3	299.371	3.866
2017	independent photographs ~ slope + distance to water + NDVI	3	299.409	3.903
2017	independent photographs ~ NDVI	1	299.419	3.913
2017	independent photographs ~ elevation + distance to settlement + distance to water	3	299.504	3.999

2018	independent photographs ~ elevation + distance to road + distance to settlement	3	340.629	0.000
2018	independent photographs ~ elevation + distance to settlement	2	341.442	0.813
2018	independent photographs ~ elevation + distance to settlement + NDVI	3	341.683	1.053
2018	independent photographs ~ elevation + NDVI	2	342.041	1.412
2018	independent photographs ~ elevation + distance to road + distance to settlement + NDVI	4	342.518	1.889
2018	independent photographs ~ elevation + distance to road + distance to settlement + distance to water	4	342.990	2.361
2018	independent photographs ~ elevation + slope + distance to road + distance to settlement	4	343.040	2.411
2018	independent photographs ~ elevation + distance to settlement + distance to water	3	343.626	2.997
2018	independent photographs ~ elevation + slope + distance to settlement	3	343.722	3.093
2018	independent photographs ~ elevation + distance to settlement + distance to water + NDVI	4	343.885	3.256
2018	independent photographs ~ elevation + slope + distance to settlement + NDVI	4	343.956	3.326
2018	independent photographs ~ elevation	1	344.093	3.464
2018	independent photographs ~ elevation + slope + NDVI	3	344.108	3.479
2018	independent photographs ~ elevation + distance to road + NDVI	3	344.296	3.667
2018	independent photographs ~ elevation + distance to water + NDVI	3	344.315	3.685
2019	independent photographs ~ elevation + distance to road + NDVI	3	298.862	0.000
2019	independent photographs ~ elevation + NDVI	2	298.977	0.115
2019	independent photographs ~ elevation + slope + NDVI	3	299.437	0.575
2019	independent photographs ~ elevation + slope + distance to road + NDVI	4	299.898	1.035
2019	independent photographs ~ elevation + distance to road + distance to water	3	300.180	1.318
2019	independent photographs ~ elevation + distance to road + distance to water + NDVI	4	300.347	1.485
2019	independent photographs ~ elevation + distance to road + distance to settlement + NDVI	4	300.750	1.888
2019	independent photographs ~ elevation + distance to water + NDVI	3	300.821	1.959

2019	independent photographs ~ elevation + slope + distance to water + NDVI	4	301.007	2.145
2019	independent photographs ~ elevation + distance to settlement + NDVI	3	301.103	2.241
2019	independent photographs ~ elevation + slope + distance to road + distance to water + NDVI	5	301.138	2.276
2019	independent photographs ~ elevation + slope + distance to road + distance to water	4	301.559	2.697
2019	independent photographs ~ elevation + slope + distance to settlement + NDVI	4	301.764	2.902
2019	independent photographs ~ elevation + distance to road + distance to settlement + distance to water	4	301.898	3.035
2019	independent photographs ~ elevation + distance to water	2	301.929	3.067
2019	independent photographs ~ elevation + distance to road + distance to settlement	3	302.001	3.139
2019	independent photographs ~ elevation + slope + distance to road + distance to settlement + NDVI	5	302.059	3.197
2019	independent photographs ~ elevation + distance to road	2	302.679	3.817
2019	independent photographs ~ elevation + distance to settlement + distance to water + NDVI	4	302.737	3.875
2019	independent photographs ~ elevation + distance to road + distance to settlement + distance to water + NDVI	5	302.748	3.886
2019	independent photographs ~ elevation + slope + distance to water	3	302.755	3.893
2020	independent photographs ~ elevation + distance to settlement + NDVI	3	261.154	0.000
2020	independent photographs ~ elevation + distance to road + distance to settlement + NDVI	4	262.322	1.168
2020	independent photographs ~ elevation + distance to road + distance to settlement	3	263.173	2.019
2020	independent photographs ~ elevation + distance to settlement + distance to water + NDVI	4	263.560	2.405
2020	independent photographs ~ elevation + distance to settlement	2	263.730	2.576
2020	independent photographs ~ elevation + slope + distance to settlement + NDVI	4	263.768	2.614
2020	independent photographs ~ elevation + distance to road + distance to settlement + distance to water + NDVI	5	265.075	3.921
2020	independent photographs ~ elevation + slope + distance to road + distance to settlement + NDVI	5	265.089	3.935

2021	independent photographs ~ elevation + distance to settlement	2	340.380	0.000
2021	independent photographs ~ elevation + distance to settlement + distance to water	3	341.238	0.858
2021	independent photographs ~ elevation + distance to road + distance to settlement	3	341.451	1.071
2021	independent photographs ~ elevation + distance to settlement + NDVI	3	341.960	1.580
2021	independent photographs ~ elevation + distance to settlement + distance to water + NDVI	4	341.990	1.610
2021	independent photographs ~ elevation + distance to road + distance to settlement + distance to water	4	342.438	2.058
2021	independent photographs ~ elevation + slope + distance to settlement	3	342.629	2.249
2021	independent photographs ~ elevation + slope + distance to settlement + distance to water	4	343.714	3.333
2021	independent photographs ~ elevation + distance to road + distance to settlement + NDVI	4	343.776	3.396
2021	independent photographs ~ elevation + slope + distance to road + distance to settlement	4	343.906	3.526
2021	independent photographs ~ elevation + distance to road + distance to settlement + distance to water + NDVI	5	344.252	3.872
2021	independent photographs ~ elevation + slope + distance to settlement + NDVI	4	344.355	3.975
2022	independent photographs ~ distance to road + distance to settlement + distance to water	3	309.576	0.000
2022	independent photographs ~ elevation + distance to road + distance to settlement + distance to water	4	310.136	0.559
2022	independent photographs ~ slope + distance to road + distance to settlement + distance to water	4	310.247	0.671
2022	independent photographs ~ elevation + distance to road + distance to settlement	3	311.153	1.577
2022	independent photographs ~ distance to road + distance to settlement + distance to water + NDVI	4	311.157	1.581
2022	independent photographs ~ elevation + slope + distance to road + distance to settlement + distance to water	5	311.230	1.654
2022	independent photographs ~ slope + distance to road + distance to settlement + distance to water + NDVI	5	311.518	1.942
2022	independent photographs ~ elevation + slope + distance to road + distance to settlement	4	312.703	3.127

2022	independent photographs ~ elevation + distance to road + distance to settlement + distance to water + NDVI	5	312.795	3.219
2022	independent photographs ~ elevation + distance to road + distance to settlement + NDVI	4	313.465	3.889
2023	independent photographs ~ elevation + distance to settlement + distance to water + NDVI	4	533.771	0.000
2023	independent photographs ~ elevation + slope + distance to settlement + distance to water + NDVI	5	535.673	1.902
2023	independent photographs ~ elevation + distance to road + distance to settlement + distance to water + NDVI	5	535.945	2.174
2023	independent photographs ~ elevation + distance to water + NDVI	3	536.895	3.124
2024	independent photographs ~ distance to settlement + distance to water	2	492.867	0.000
2024	independent photographs ~ distance to water	1	492.960	0.093
2024	independent photographs ~ distance to water + NDVI	2	493.605	0.738
2024	independent photographs ~ slope + distance to settlement + distance to water	3	493.650	0.782
2024	independent photographs ~ elevation + distance to water + NDVI	3	494.155	1.288
2024	independent photographs ~ distance to settlement + distance to water + NDVI	3	494.471	1.604
2024	independent photographs ~ distance to road + distance to settlement + distance to water	3	494.545	1.677
2024	independent photographs ~ slope + distance to water	2	494.653	1.785
2024	independent photographs ~ slope + distance to water + NDVI	3	494.796	1.929
2024	independent photographs ~ slope + distance to settlement + distance to water + NDVI	4	494.812	1.944
2024	independent photographs ~ elevation + slope + distance to settlement + distance to water + NDVI	5	494.880	2.013
2024	independent photographs ~ slope + distance to road + distance to settlement + distance to water	4	494.886	2.019
2024	independent photographs ~ elevation + distance to settlement + distance to water	3	494.918	2.051
2024	independent photographs ~ elevation + distance to settlement + distance to water + NDVI	4	494.922	2.054
2024	independent photographs ~ distance to road + distance to water	2	495.117	2.250
2024	independent photographs ~ elevation + distance to water	2	495.119	2.252

2024	independent photographs ~ elevation + slope + distance to water + NDVI	4	495.183	2.316
2024	independent photographs ~ distance to road + distance to water + NDVI	3	495.609	2.742
2024	independent photographs ~ elevation + slope + distance to settlement + distance to water	4	495.857	2.990
2024	independent photographs ~ elevation + distance to road + distance to settlement + distance to water	4	496.037	3.170
2024	independent photographs ~ elevation + distance to road + distance to water + NDVI	4	496.321	3.454
2024	independent photographs ~ elevation + slope + distance to road + distance to settlement + distance to water	5	496.353	3.486
2024	independent photographs ~ distance to road + distance to settlement + distance to water + NDVI	4	496.699	3.831
2024	independent photographs ~ slope + distance to road + distance to water + NDVI	4	496.788	3.921
2024	independent photographs ~ elevation + slope + distance to road + distance to settlement + distance to water + NDVI	6	496.806	3.939

Table S16. Model- averaged results for exploring the relationships between numbers of independent photographs of sika deer and explanatory variables (distance to road, distance to settlement, distance to water, elevation, slope, year, Normalized Difference Vegetation Index, and Leaf Area Index) from 2015 to 2024. Leaf Area Index and Normalized Difference Vegetation Index were modeled separately in optimal models. Models with ΔAICc < 4 were selected and averaged.

Models	Explanatory variables	Estimate	SE	P
LAI				
2015	Intercept	4.071	0.572	0.000
2015	Elevation	0.847	0.822	0.313
2015	Slope	0.251	0.524	0.642
2015	Distance to road	0.332	0.647	0.619
2015	Distance to settlement	-1.590	0.616	0.012
2015	Distance to water	1.314	0.423	0.003
2015	LAI	-2.985	0.836	0.001
2016	Intercept	3.751	0.568	0.000

2016	Elevation	1.118	0.533	0.042
2016	LAI	-3.337	0.699	0.000
2016	Distance to water	0.526	0.494	0.299
2016	Distance to settlement	-0.367	0.615	0.564
2016	Slope	0.253	0.485	0.614
2016	Distance to road	-0.010	0.485	0.984
2017	Intercept	2.640	0.668	0.000
2017	Elevation	1.039	0.585	0.086
2017	Slope	0.559	0.527	0.306
2017	Distance to road	0.009	0.498	0.986
2017	Distance to settlement	-0.420	0.617	0.512
2017	Distance to water	0.548	0.493	0.280
2017	LAI	-0.844	0.748	0.276
2018	Intercept	1.664	0.526	0.002
2018	Elevation	2.749	0.559	0.000
2018	Slope	-0.004	0.513	0.994
2018	Distance to road	1.042	0.594	0.089
2018	Distance to settlement	-1.643	0.700	0.022
2018	Distance to water	0.149	0.467	0.757
2018	LAI	-0.755	0.739	0.320
2019	Intercept	1.736	0.955	0.074
2019	Elevation	3.375	0.783	0.000
2019	Slope	-0.742	0.538	0.184
2019	Distance to road	0.693	0.547	0.217
2019	Distance to settlement	-0.482	0.688	0.494
2019	Distance to water	-0.713	0.511	0.175
2019	LAI	-1.267	0.653	0.061
2020	Intercept	2.282	0.592	0.000
2020	Elevation	2.725	0.548	0.000
2020	Slope	0.146	0.468	0.765
2020	Distance to road	1.018	0.773	0.200
2020	Distance to settlement	-1.664	0.814	0.046
2020	Distance to water	-0.120	0.444	0.794
2020	LAI	-1.149	0.646	0.087
2021	Intercept	2.415	0.432	0.000
2021	Elevation	2.180	0.562	0.000
2021	Slope	-0.124	0.575	0.835
2021	Distance to road	0.822	0.691	0.250
2021	Distance to settlement	-1.736	0.649	0.009
2021	Distance to water	0.590	0.456	0.211
2021	LAI	-0.046	0.831	0.957
2022	Intercept	3.741	0.408	0.000
2022	Elevation	0.883	0.537	0.111
2022	Slope	0.641	0.448	0.171
2022	Distance to road	2.124	0.717	0.004
2022	Distance to settlement	-4.238	0.951	0.000
2022	Distance to water	1.092	0.399	0.008
2022	LAI	-0.514	0.483	0.308
2023	Intercept	4.080	0.495	0.000

2023	Elevation	-0.777	0.690	0.268
2023	Slope	-0.567	0.394	0.159
2023	Distance to road	0.756	0.518	0.153
2023	Distance to settlement	-1.268	0.524	0.018
2023	Distance to water	1.102	0.420	0.010
2023	LAI	-0.388	1.021	0.707
2024	Intercept	3.425	0.288	0.000
2024	Elevation	-0.187	0.481	0.705
2024	Slope	0.428	0.398	0.294
2024	Distance to road	0.335	0.538	0.541
2024	Distance to settlement	-0.723	0.433	0.103
2024	Distance to water	1.276	0.331	0.000
2024	LAI	-0.023	0.365	0.951
NDVI				
2015	Intercept	2.192	0.446	0.000
2015	Elevation	1.768	0.587	0.003
2015	Slope	0.326	0.553	0.568
2015	Distance to road	1.043	0.661	0.125
2015	Distance to settlement	-2.541	0.738	0.001
2015	Distance to water	0.362	0.474	0.458
2015	NDVI	0.328	0.551	0.562
2016	Intercept	2.324	0.429	0.000
2016	Elevation	1.509	0.571	0.010
2016	Slope	0.114	0.531	0.836
2016	Distance to road	1.146	0.685	0.106
2016	Distance to settlement	-2.083	0.796	0.011
2016	Distance to water	0.262	0.513	0.621
2016	NDVI	-0.324	0.569	0.582
2017	Intercept	2.529	0.599	0.000
2017	Elevation	1.068	0.567	0.069
2017	Slope	0.578	0.527	0.291
2017	Distance to road	0.009	0.498	0.986
2017	Distance to settlement	-0.332	0.601	0.595
2017	Distance to water	0.537	0.497	0.293
2017	NDVI	-0.745	0.806	0.371
2018	Intercept	1.722	0.497	0.001
2018	Elevation	2.692	0.567	0.000
2018	Slope	0.109	0.549	0.847
2018	Distance to road	0.938	0.669	0.171
2018	Distance to settlement	-1.523	0.732	0.041
2018	Distance to water	0.156	0.468	0.746
2018	NDVI	-0.974	0.632	0.133
2019	Intercept	2.077	1.074	0.057
2019	Elevation	3.072	0.858	0.000
2019	Slope	-0.833	0.537	0.136
2019	Distance to road	0.73	0.468	0.132
2019	Distance to settlement	-0.297	0.74	0.695
2019	Distance to water	-0.678	0.497	0.186
2019	NDVI	-1.534	0.679	0.029

2020	Intercept	2.736	0.751	0.000
2020	Elevation	2.588	0.525	0.000
2020	Slope	-0.048	0.425	0.914
2020	Distance to road	1.001	0.656	0.143
2020	Distance to settlement	-1.558	0.715	0.034
2020	Distance to water	0.203	0.396	0.624
2020	NDVI	-1.396	0.687	0.052
2021	Intercept	2.509	0.455	0.000
2021	Elevation	2.125	0.567	0.000
2021	Slope	-0.124	0.575	0.835
2021	Distance to road	0.778	0.694	0.279
2021	Distance to settlement	-1.693	0.639	0.010
2021	Distance to water	0.627	0.462	0.189
2021	NDVI	-0.544	0.633	0.406
2022	Intercept	3.706	0.379	0.000
2022	Elevation	0.918	0.53	0.093
2022	Slope	0.642	0.45	0.172
2022	Distance to road	2.112	0.721	0.005
2022	Distance to settlement	-4.224	0.954	0.000
2022	Distance to water	1.107	0.405	0.008
2022	NDVI	-0.528	0.508	0.319
2023	Intercept	6.01	0.638	0.000
2023	Elevation	-1.896	0.611	0.002
2023	Slope	-0.248	0.362	0.504
2023	Distance to road	0.259	0.469	0.589
2023	Distance to settlement	-1.098	0.418	0.010
2023	Distance to water	1.837	0.403	0.000
2023	NDVI	-2.435	0.66	0.000
2024	Intercept	3.652	0.525	0.000
2024	Elevation	-0.535	0.632	0.407
2024	Slope	0.45	0.398	0.271
2024	Distance to road	0.201	0.567	0.729
2024	Distance to settlement	-0.686	0.433	0.122
2024	Distance to water	1.294	0.352	0.000
2024	NDVI	-0.832	0.668	0.222
